# Supplementary material for: A neomorphic variant in SP7 alters sequence specificity and causes a high-turnover bone disorder
Source: Nat Commun. 2022 Feb 4;13:700. doi: 10.1038/s41467-022-28318-4 (PMC8816926; doi:10.1038/s41467-022-28318-4)
Supplement: Supplementary file 1 — Supplementary Information [file 41467_2022_28318_MOESM1_ESM.pdf]

Supplementary information

Supplement to: “A Neomorphic Variant in SP7 Alters Sequence Specificity and Causes a High-Turnover Bone Disorder”

Content: Page

Supplementary Figure 1	2
Supplementary Figure 2	3
Supplementary Figure 3	4
Supplementary Figure 4	5
Supplementary Figure 5	6
Supplementary Figure 6	7
Supplementary Figure 7	8
Supplementary Figure 8	9
Supplementary Figure 9	10
Supplementary Figure 10	11
Supplementary Figure 11	12
Supplementary Figure 12	13
Supplementary Figure 13	14
Supplementary References	15

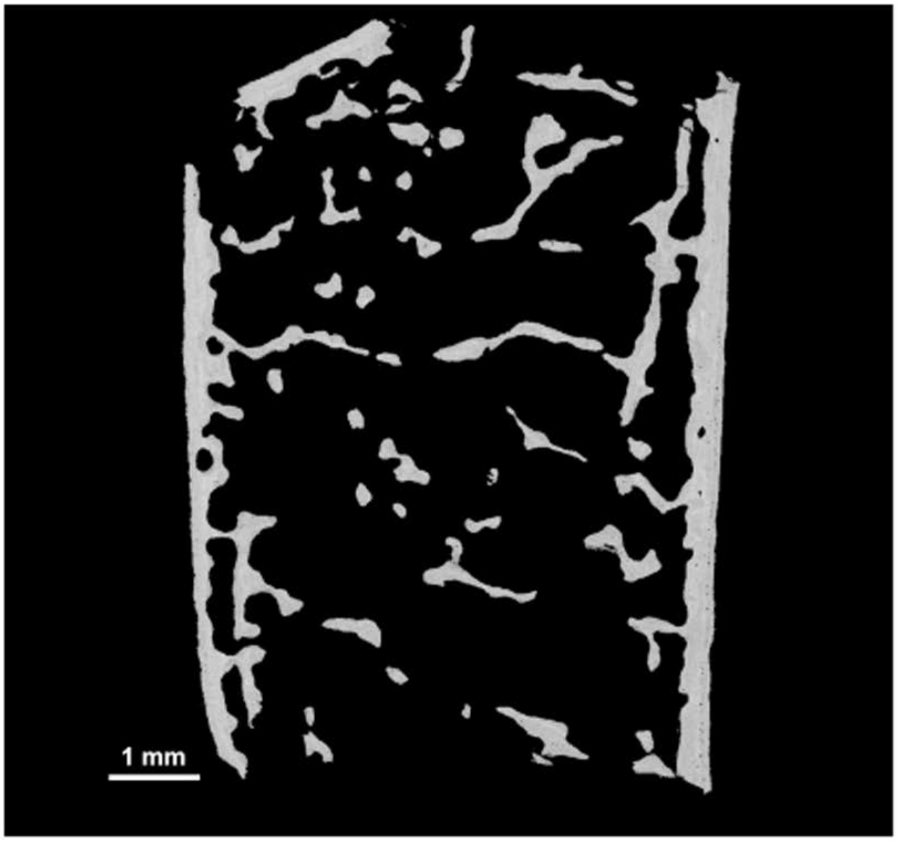

Structural indices	Patient (3 yrs, male)	Reference
BV/TV (%)	13.24	17.7 ± 2.6
md. BV/TV (%)	12.32	
Tb.Th. (μm)	144.73	101 ± 11
md. Tb.Th. (μm)	108	
Tb.N (mm^{-1})		1.77 ± 0.31
md. Tb.N (mm^{-1})	1.14	
Ct.Wi. (mm)	0.33	0.70 ± 0.28
Ct. Po (%)	0.92	

m.d. = mineralized bone matrix only

Supplementary Figure 1. Backscattered electron microscopy image of the biopsy sample, structural indices of bone histomorphometry, compared to age-matched reference range. The data was obtained from a single transiliac bone biopsy from the patient, using the entire cross-sectioned area of the residual sample block for analysis, following standardized procedures for histology and histomorphometry and using the entire cross-sectioned area[1].

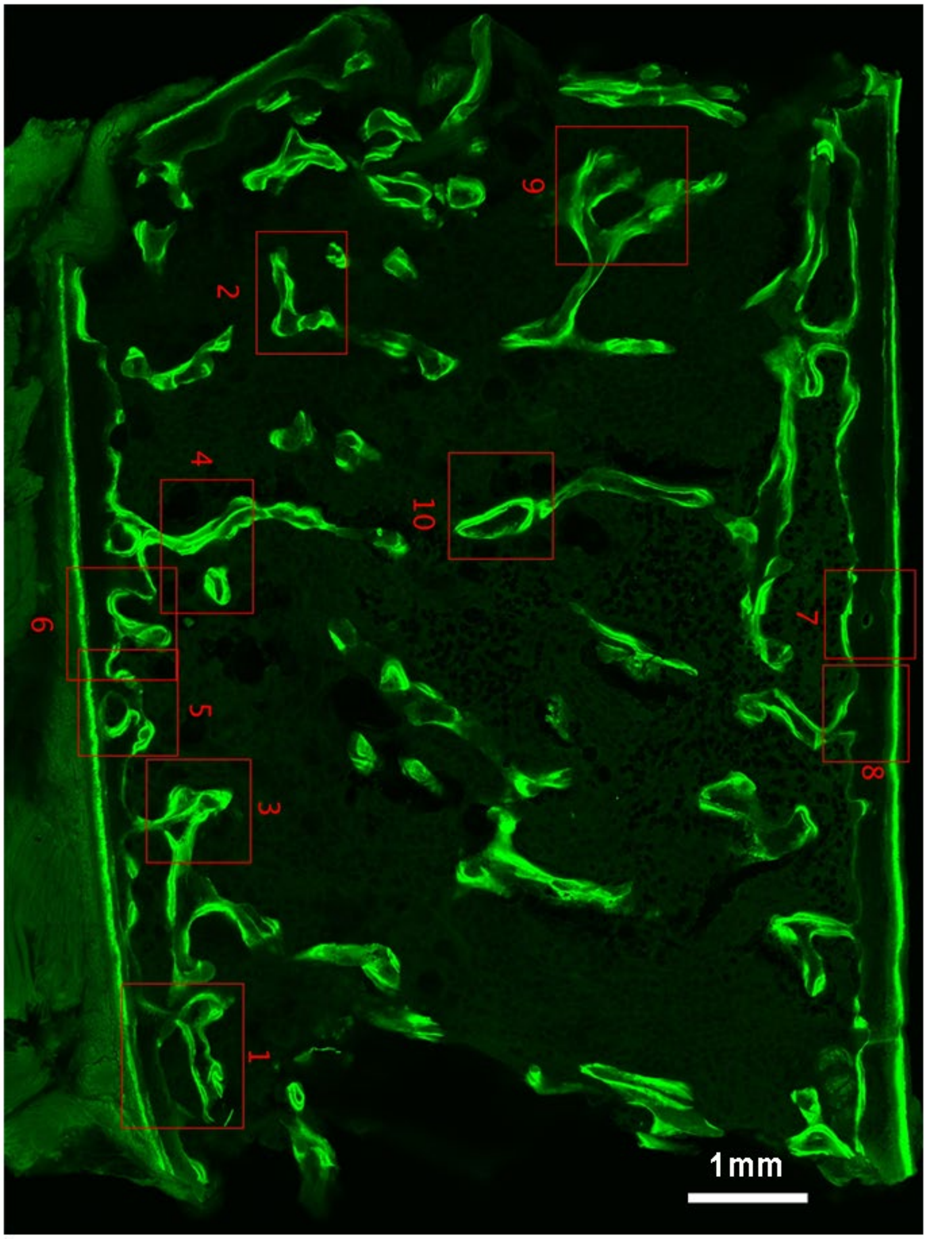

dynamic indices of bone formation	Patient	*Reference[1]*
MS/BS [%]	16.03	*12.5 ± 4.5*
MAR [μm d^{-1}]	1.00	*1.04 ± 0.17*
BFR/BS [μm^3μm^{-2}a^{-1}]	58.5	*48.1 ± 19.4*

Supplementary Figure 2. Dynamic fluorescent bone imaging after tetracycline labeling and quantified dynamic indices measurements, compared to age-matched reference range[1]. Red boxes indicate area where the measurements were taken. The data was obtained from a single transiliac bone biopsy from the patient.

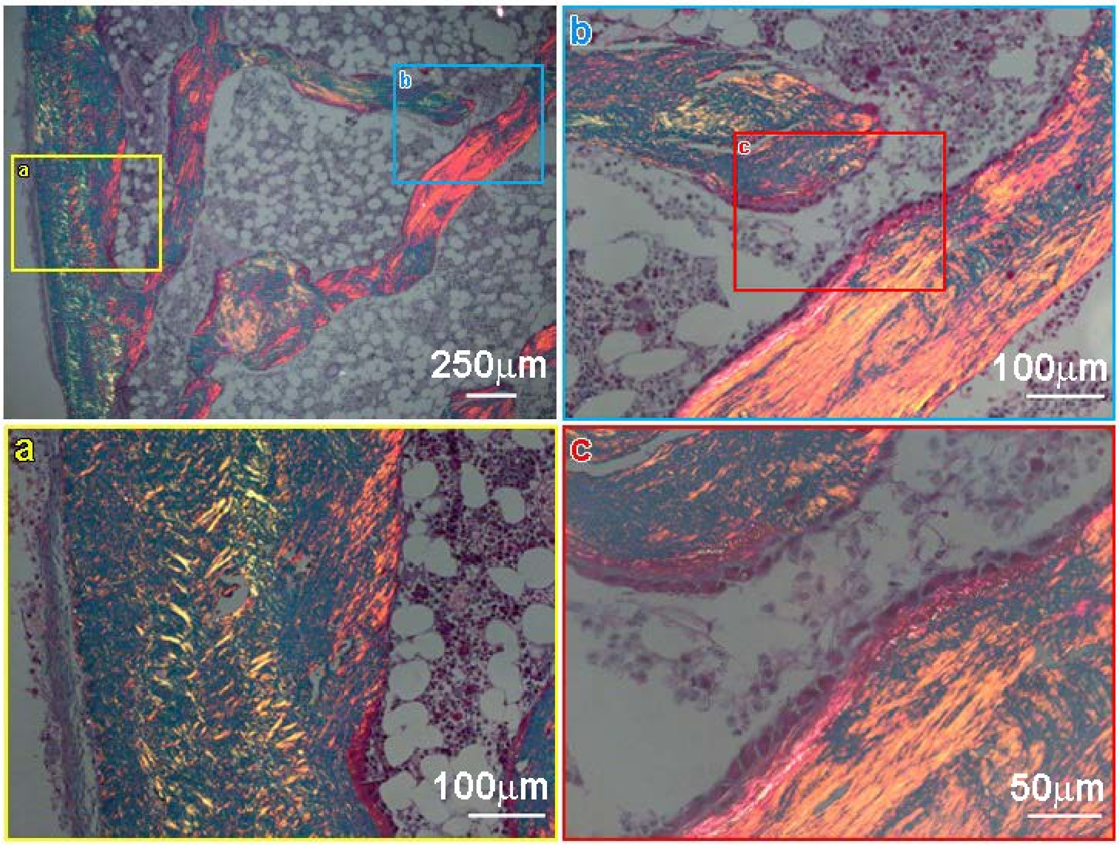

Supplementary Figure 3. Polarized light microscopy showing abnormal collagen fibril organization in the bone biopsy sample. a, b, and c showed the enlarged view of the original image. The data was obtained from a single transiliac bone biopsy from the patient.

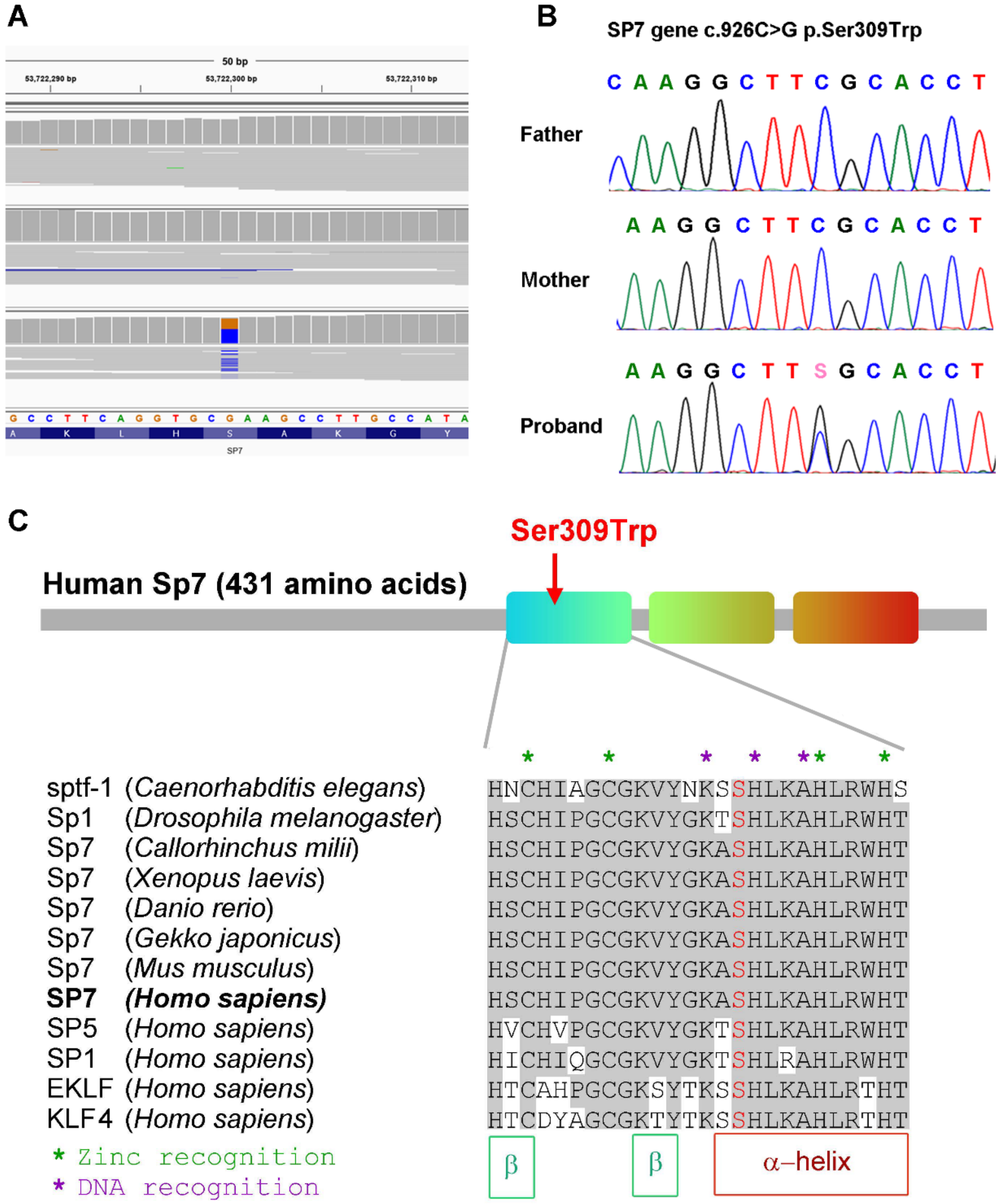

Supplementary Figure 4. (A) Exome sequencing identified a heterozygous base-pair substitution in the proband but neither parent. Orange rectangle indicates G (variant sequence); blue rectangle indicates presence of C (reference sequence); gray rectangle in parents at the same nucleotide also indicates presence of C (reference sequence). (B) Sanger sequencing confirmed the presence of the C > G substitution in the proband and the absence in the parents. (C) Evolutionary conservation of the first zinc finger motif of SP7, where the S309W variant is located, across different species, and between different Krüppel-like family of transcription factors.

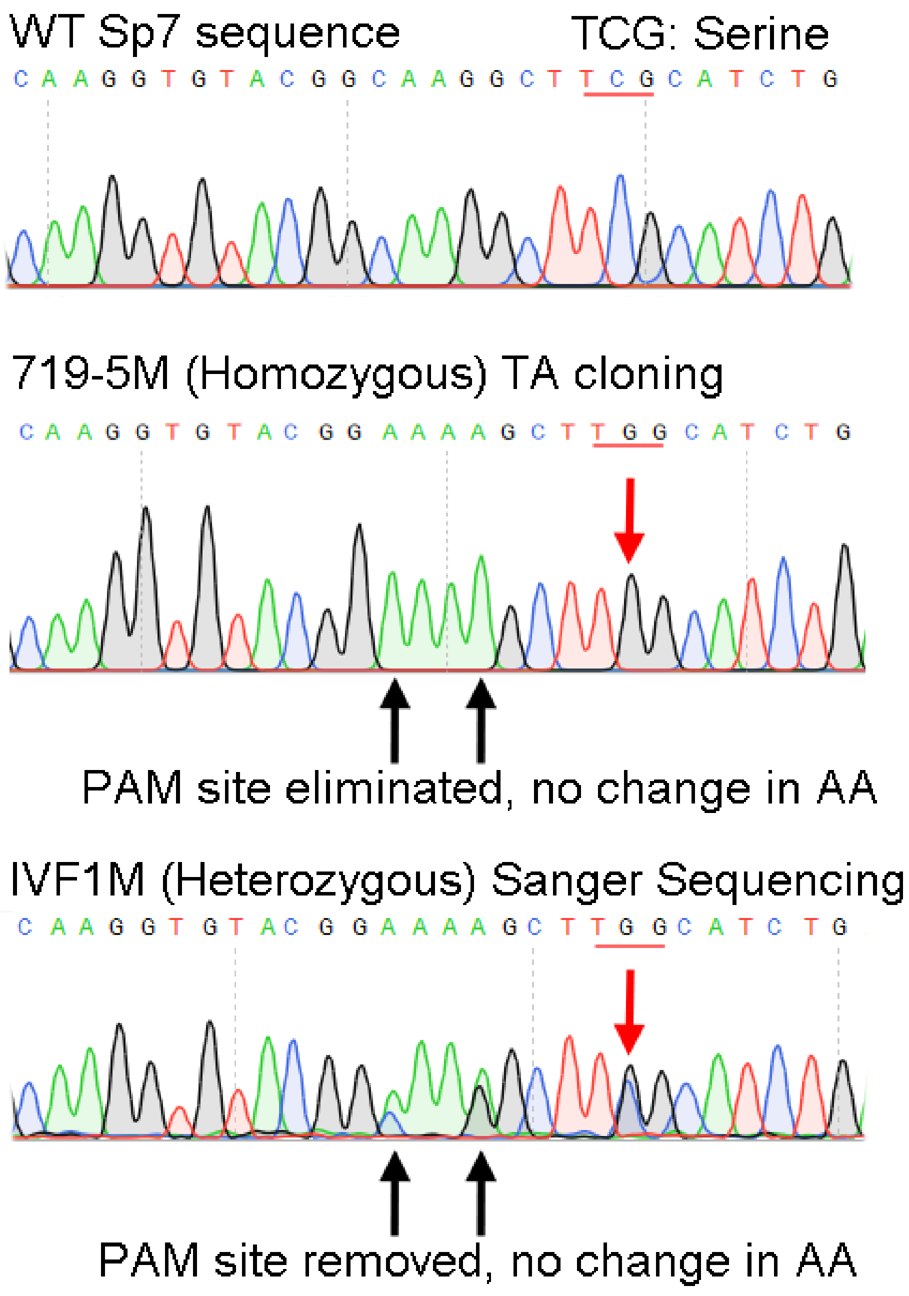

Supplementary Figure 5. Sanger sequencing (from PCR or TA cloned PCR product) confirming the SP7 homozygous and heterozygous mutant mice. The codon for amino acid 309 was underlined in red and indicated by a red arrow. Black arrows indicate two synonymous mutations intentionally introduced into the mice to eliminate the PAM site used by CRISPR enzyme, to terminate further genome editing once the correct mutation was introduced.

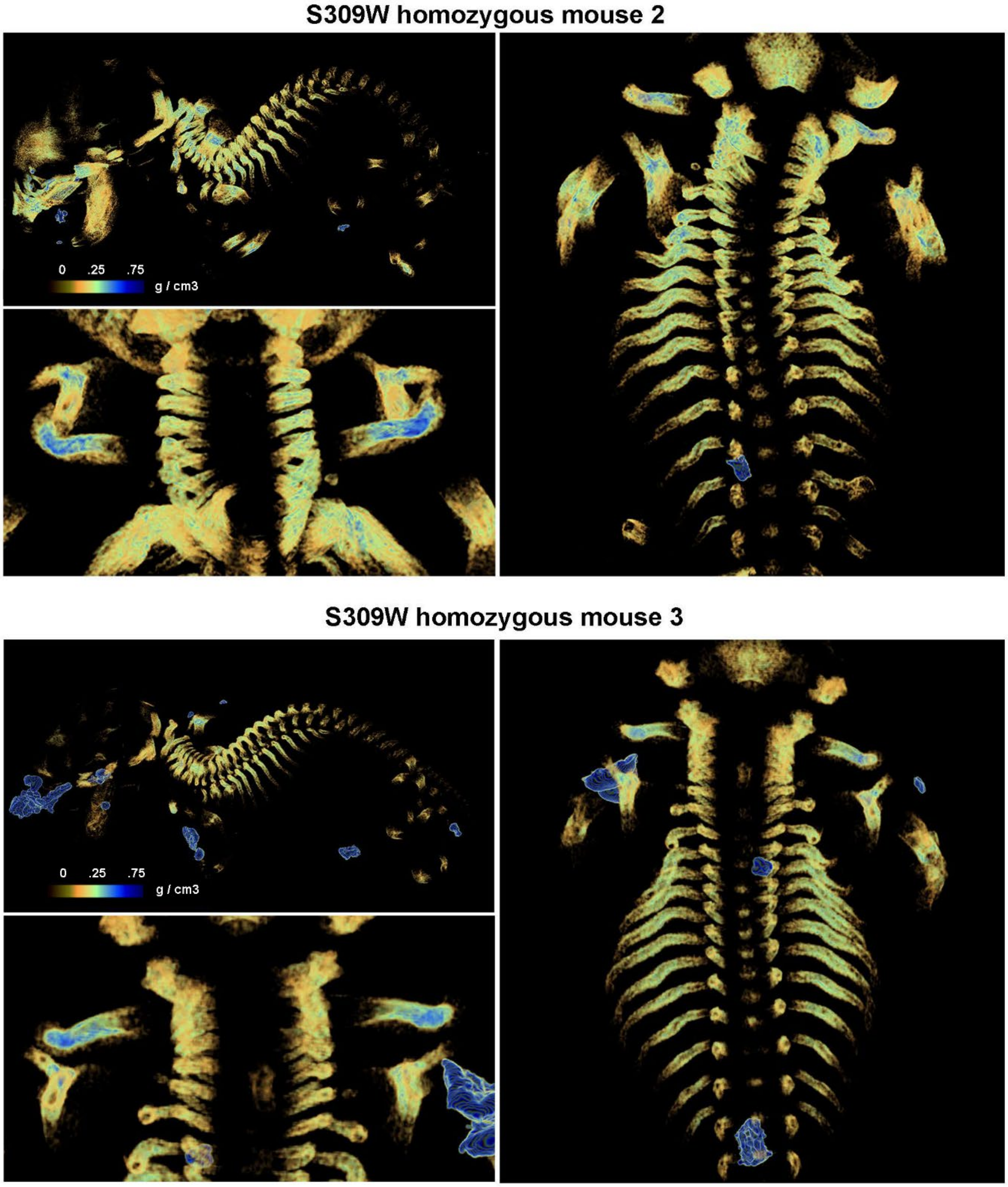

Supplementary Figure 6. Micro-CT images of homozygous knock-in mice not included in Figure 2, showing similar clavicle hyperostosis and abnormal rib bone thickening.

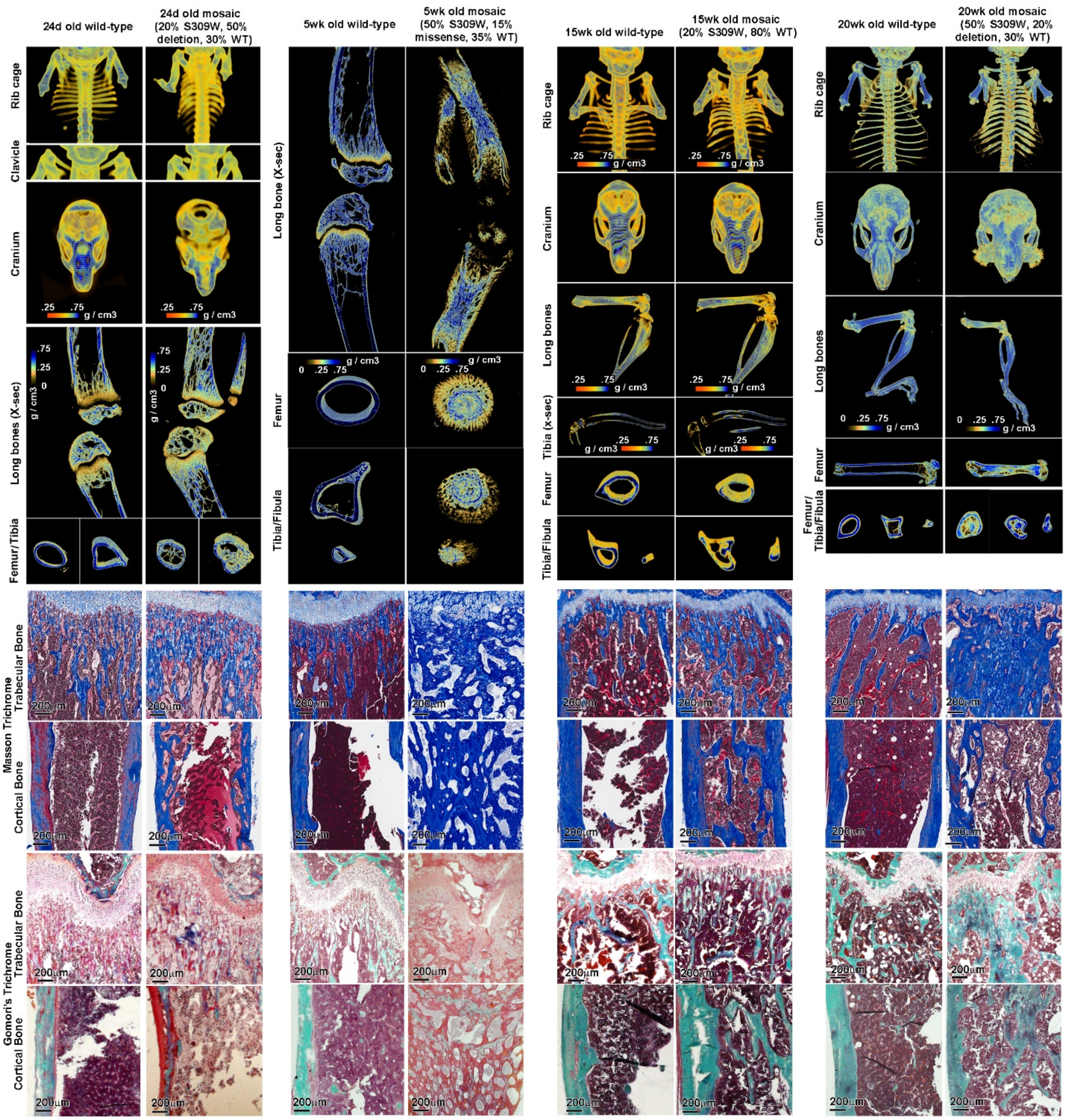

Supplementary Figure 7. Four mosaic mice at different ages (24d, 5-weeks, 15-weeks, and 20-weeks) carrying S309W mutation were analyzed by micro-CT imaging and bone histology (compared to age-matched wild-type). The mouse that died at 5 weeks was partially cannibalized and only the long bones were preserved and studied. In general, the mosaic mice commonly showed thickened rib bones and clavicles, shortened and widened long bones with decreased or complete absence of medullary cavity and uneven cortical thickness. For Masson's-Trichrome and Gomori's Trichrome staining, five histology sections were prepared and representative images were shown.

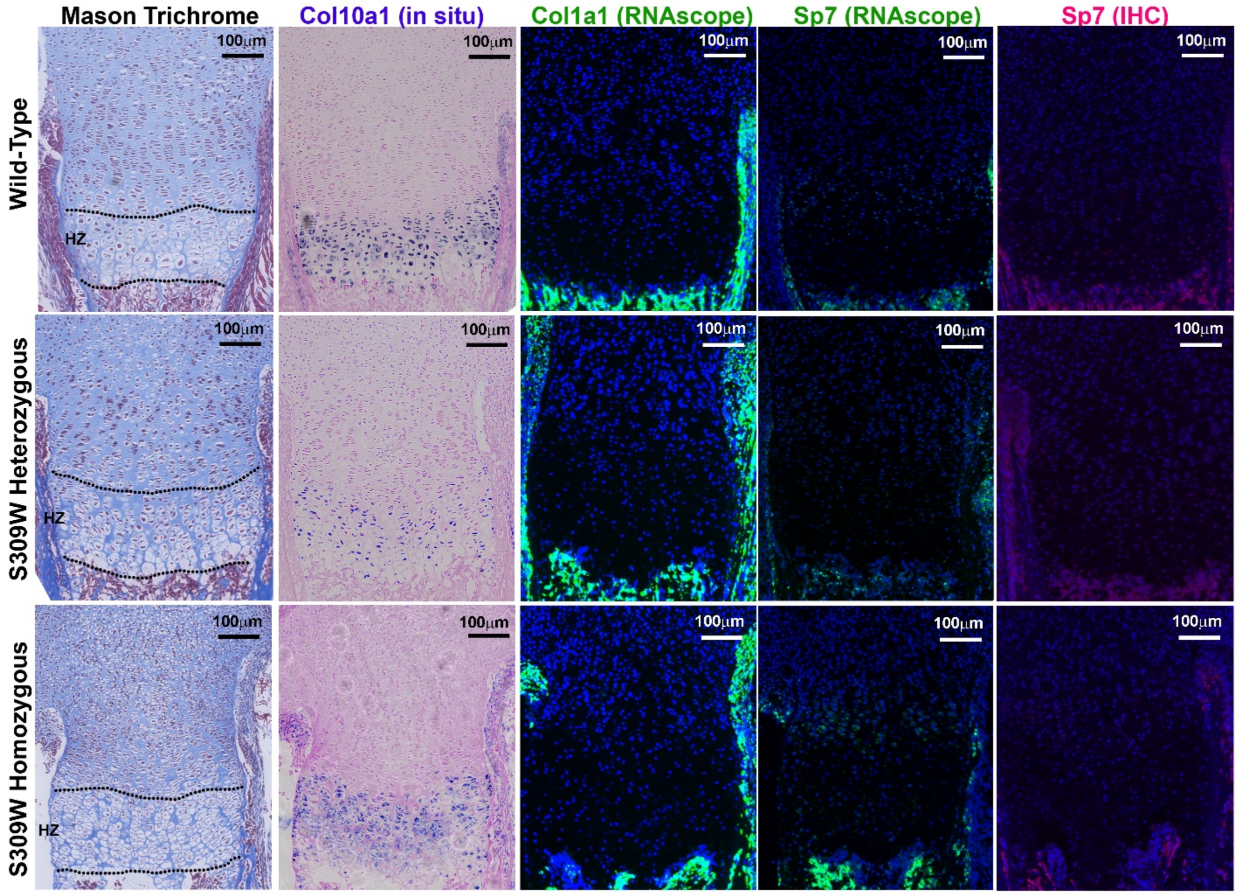

Supplementary Figure 8. Histology in the proximal tibial growth plate were similar between newborn wild-type, heterozygous and homozygous Sp7 mutants, shown by Masson's trichrome staining. In situ hybridization suggested a normal distribution of Col10a1, a marker for hypertrophic differentiation. Col1a1 mRNA expression by RNAscope and Sp7 protein expression by immunohistochemistry were detected in the perichondrium and primary spongiosa, but not in growth plate chondrocytes, with similar patterns observed in wild-type mice and those carrying the mutation. Experiment was repeated independently 3 times with similar results and representative images were shown.

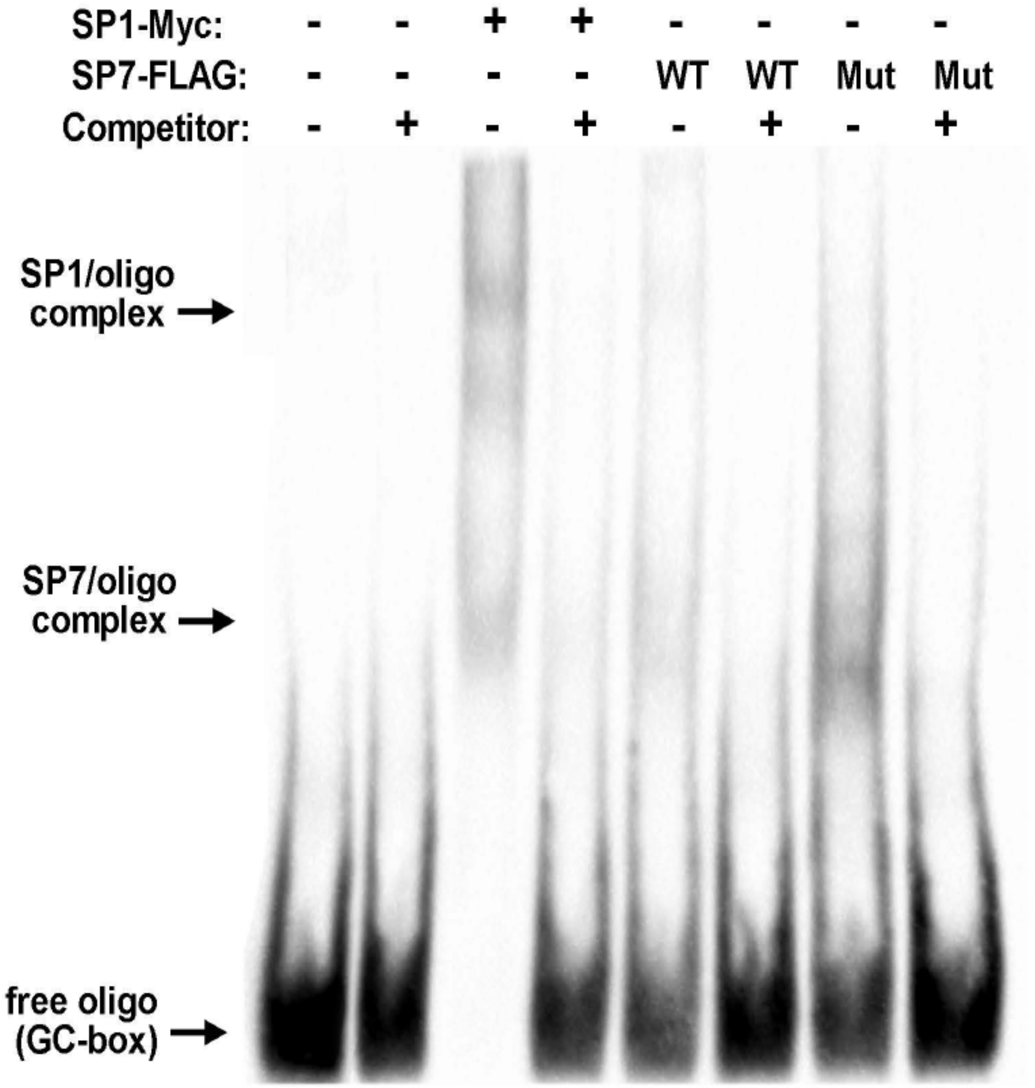

Supplementary Figure 9. Electrophoretic mobility shift assay (EMSA) in HEK293 showing interactions of SP1, wildtype SP7 and mutant SP7 with a GC-box motif, similar to that shown in Figure 4B. Unlike Figure 4B, supershift assays were not performed. Instead, non-biotinylated GCbox probe was used to compete for the binding between the tested protein and the biotinylated probe (see lane 2,4,6, and 8). Experiment was repeated independently 3 times with similar results.

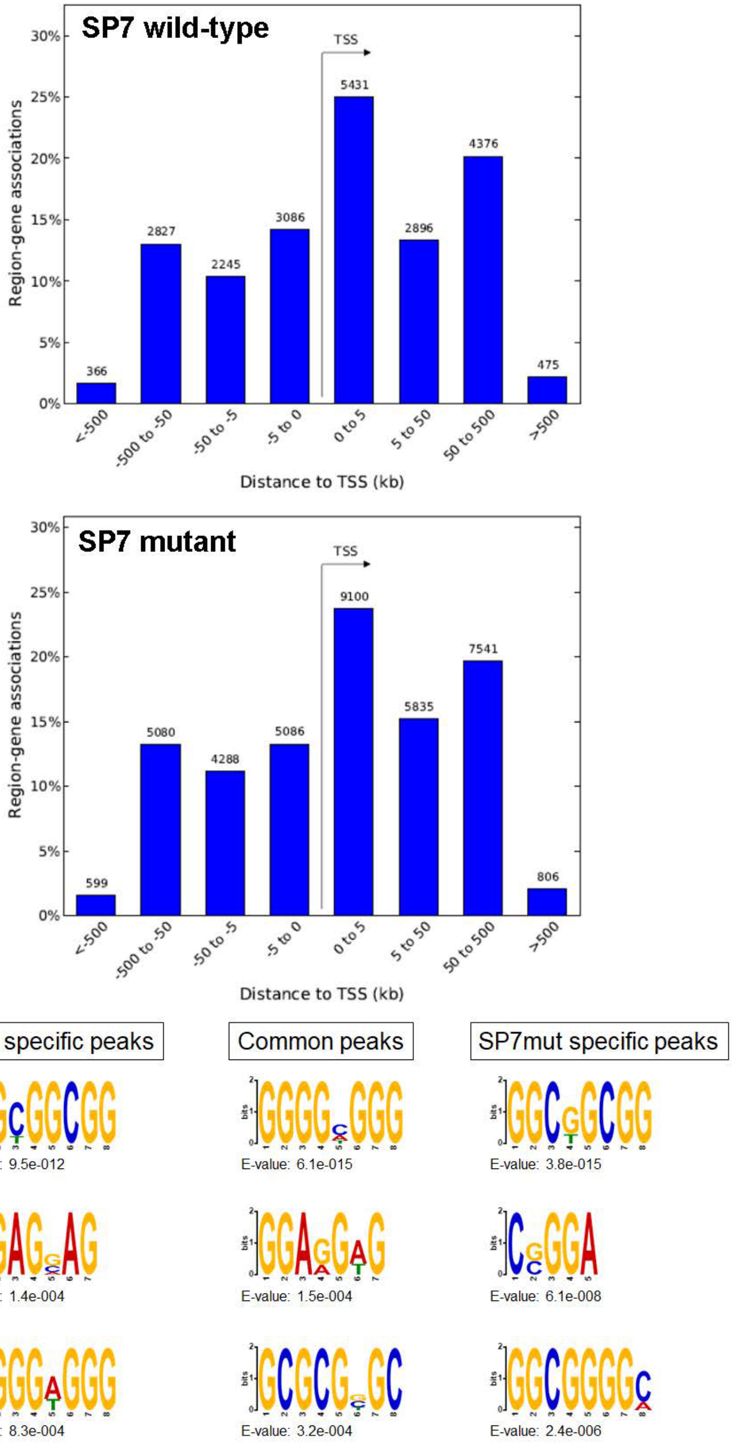

Supplementary Figure 10. Analysis of genomic binding for wild-type SP7 and S309W variant (mutant) by ChIP-Seq in primary chondrocytes. Analysis of wild-type-specific and mutant specific peaks showed that they have similar binding position in regard to proximity to transcription start sites (TSS) and binding motifs.

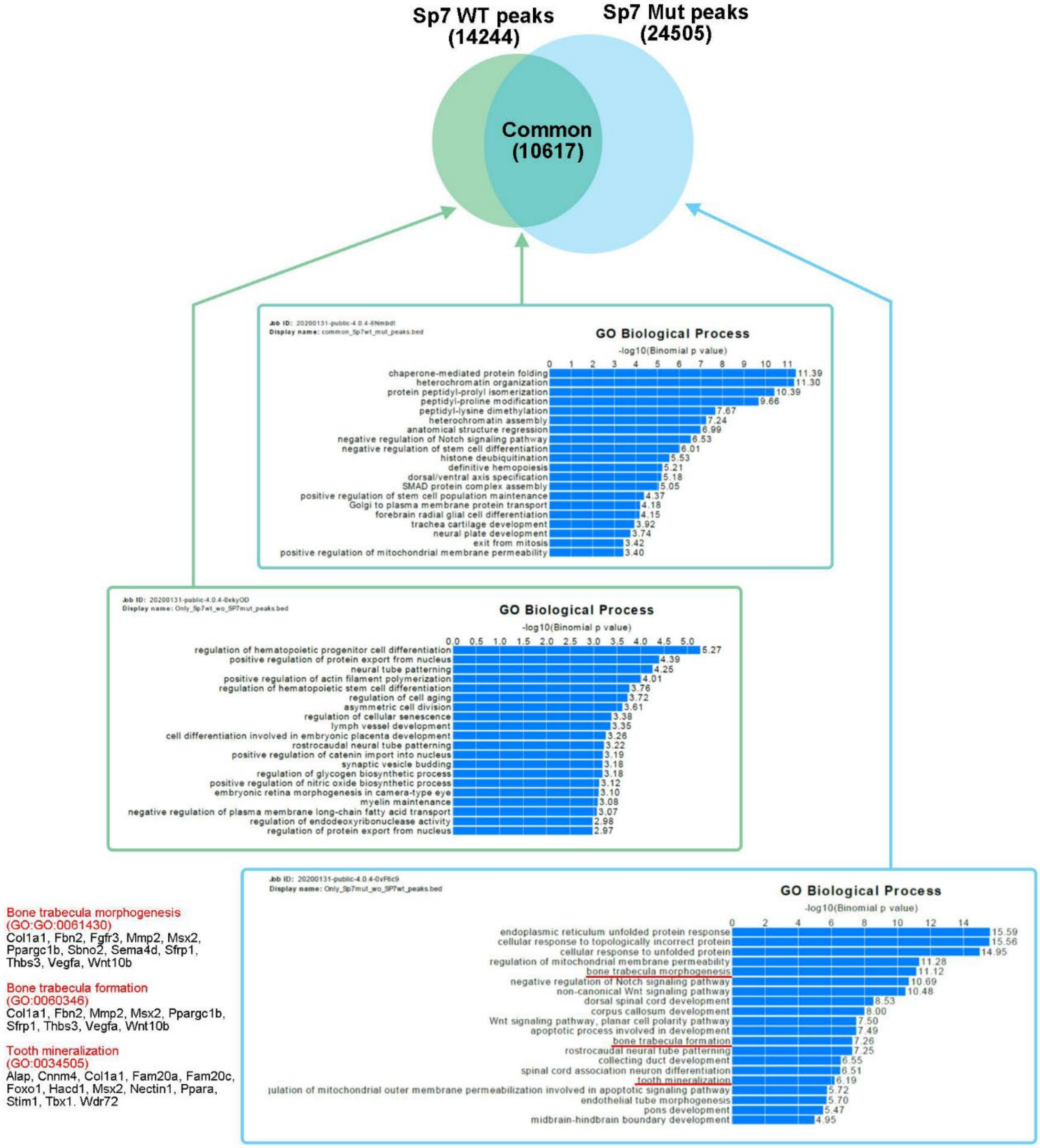

Supplementary Figure 11. Gene ontology analyses of wild-type-specific peaks, common peaks, and S309W-specific peaks in ChIP-Seq. P-values were generated with 2-sided test, with Bonferroni correction for multiple testing.

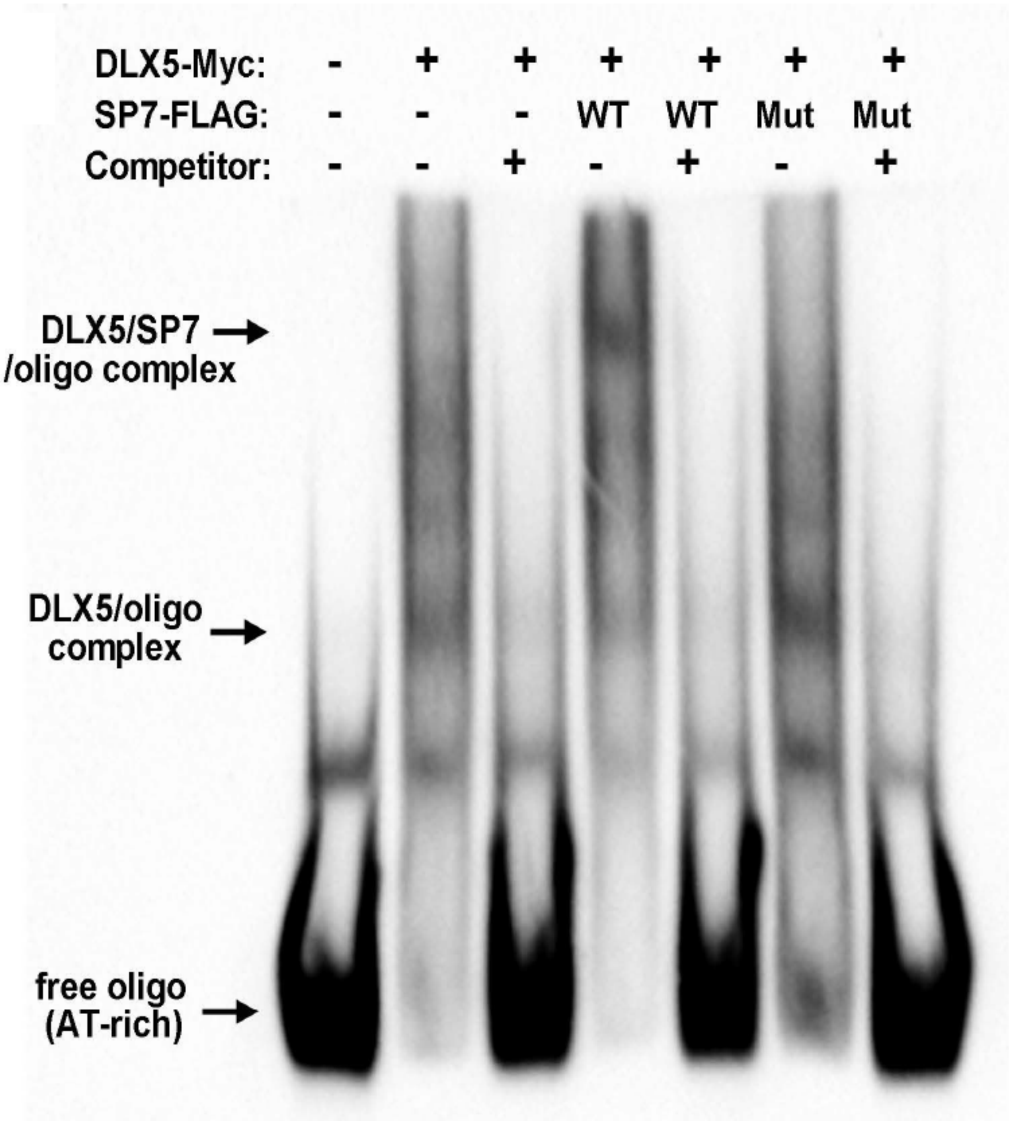

Supplementary Figure 12 Electrophoretic mobility shift assay (EMSA) in HEK293 showing interactions of DLX5 and wildtype SP7 or mutant SP7, with an AT-rich motif probe, similar to that shown in Figure 4G. Unlike Figure 4B, supershift assays were not performed. Instead, nonbiotinylated AT-rich probe was used to compete for the binding between the tested protein and the biotinylated probe (see lane 3,5 and 7). Experiment was repeated independently 3 times with similar results.

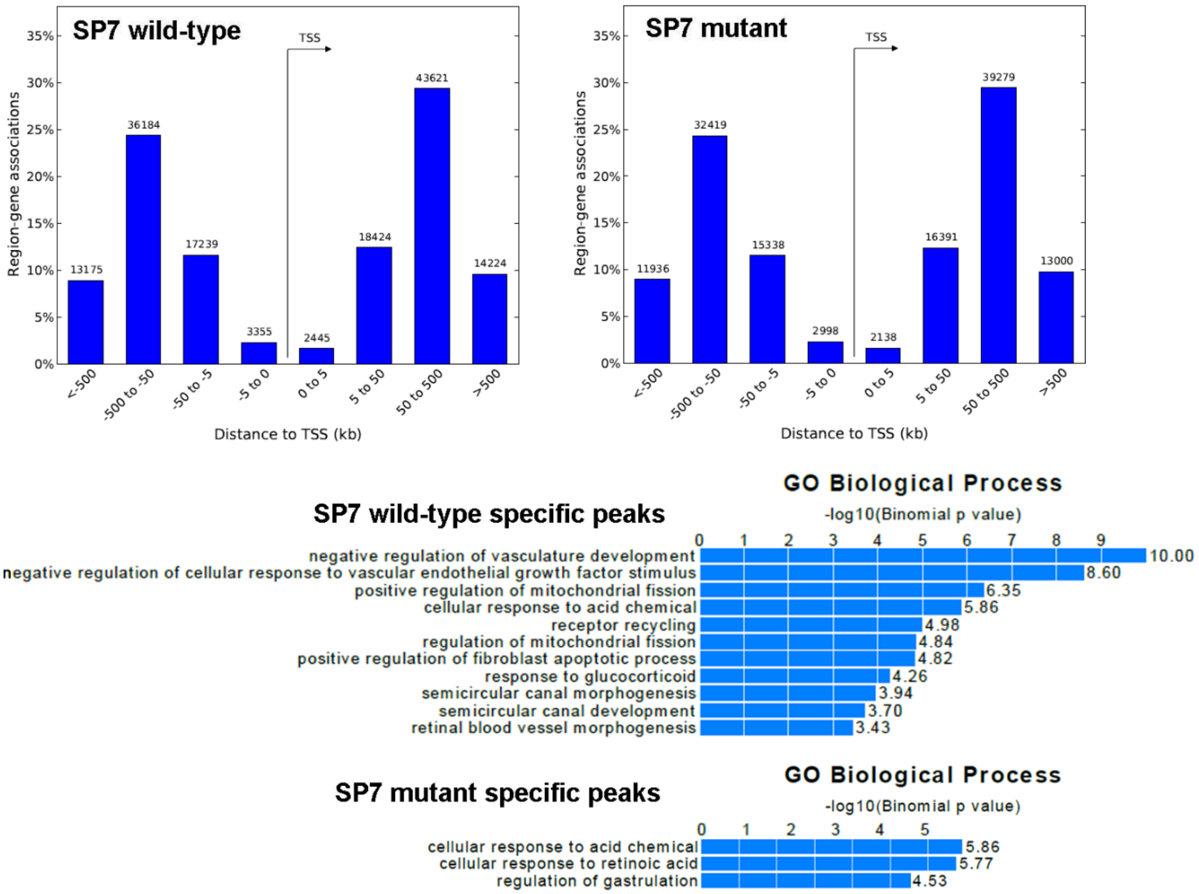

Supplementary Figure 13. Analysis of genomic binding and gene ontology functions for wild-type SP7 and S309W variant (mutant) by ChIP-Seq in MC3T3 cells expressing myc-DLX5. P-values were generated with 2-sided test, with Bonferroni correction for multiple testing Analysis of wildtype-specific and mutant-specific peaks showed that they have similar binding position in regard to proximity to transcription start sites (TSS) but different from that observed in primary chondrocytes (Figure S10).

References:

1. Glorieux, F.H. *et al.* Normative data for iliac bone histomorphometry in growing children. *Bone* **26**, 103-9 (2000).
