# Supplementary material for: A neomorphic variant in SP7 alters sequence specificity and causes a high-turnover bone disorder
Source: Nat Commun. 2022 Feb 4;13:700. doi: 10.1038/s41467-022-28318-4 (PMC8816926; doi:10.1038/s41467-022-28318-4)
Supplement: Supplementary file 2 — Reporting Summary [file 41467_2022_28318_MOESM2_ESM.pdf]

nature research

Corresponding author(s): Julian Lui

Last updated by author(s): 2022/01/03

Reporting Summary

Nature Research wishes to improve the reproducibility of the work that we publish. This form provides structure for consistency and transparency in reporting. For further information on Nature Research policies, see our Editorial Policies and the Editorial Policy Checklist.

Statistics

For all statistical analyses, confirm that the following items are present in the figure legend, table legend, main text, or Methods section.

n/a	Confirmed	
☐	☒	The exact sample size (*n*) for each experimental group/condition, given as a discrete number and unit of measurement
☐	☒	A statement on whether measurements were taken from distinct samples or whether the same sample was measured repeatedly
☐	☒	The statistical test(s) used AND whether they are one- or two-sided<br>*Only common tests should be described solely by name; describe more complex techniques in the Methods section.*
☐	☒	A description of all covariates tested
☐	☒	A description of any assumptions or corrections, such as tests of normality and adjustment for multiple comparisons
☐	☒	A full description of the statistical parameters including central tendency (e.g. means) or other basic estimates (e.g. regression coefficient) AND variation (e.g. standard deviation) or associated estimates of uncertainty (e.g. confidence intervals)
☐	☒	For null hypothesis testing, the test statistic (e.g. *F*, *t*, *r*) with confidence intervals, effect sizes, degrees of freedom and *P* value noted<br>*Give P values as exact values whenever suitable.*
☐	☒	For Bayesian analysis, information on the choice of priors and Markov chain Monte Carlo settings
☐	☒	For hierarchical and complex designs, identification of the appropriate level for tests and full reporting of outcomes
☐	☒	Estimates of effect sizes (e.g. Cohen's *d*, Pearson's *r*), indicating how they were calculated

Our web collection on statistics for biologists contains articles on many of the points above.

Software and code

Policy information about availability of computer code

Data collection	Patient bone biopsy histomorphometry image acquisition (Zeiss AxioCam videocamera), patient bone biopsy qBEI (digital scanning electron microscope DSM 962, Zeiss), Backscattered electrons measurement (four-quadrant semiconductor backscattered electron detector), mouse micro-CT (Bruker Skyscan 1172, 1272, 1176 suite, PerkinElmer, Quantum GX), western blot (Amersham Imager 680), qPCR (Applied biosystems ABI QuantStudio 6 Flex System), ChIP-Seq (illumina Hiseq X)
Data analysis	Patient bone biopsy (NIH Image software version 1.63). For micro-CT in mice, 3-Dimensional reconstruction of scanned datasets was performed using NRecon (Bruker, Billerica, MA) and rotated (only for long bones) with DataViewer (Bruker). Whole body µCT images and videos were generated by CTVox (Bruker). For study of gene expression in vitro, quantitative real-time PCR data were analyzed using Sigma Plot 11.

For manuscripts utilizing custom algorithms or software that are central to the research but not yet described in published literature, software must be made available to editors and reviewers. We strongly encourage code deposition in a community repository (e.g. GitHub). See the Nature Research guidelines for submitting code & software for further information.

Data

Policy information about availability of data

All manuscripts must include a data availability statement. This statement should provide the following information, where applicable:

- Accession codes, unique identifiers, or web links for publicly available datasets
- A list of figures that have associated raw data
- A description of any restrictions on data availability

The datasets generated during and/or analysed during the current study are available as source data file 1 (raw values from dot plots in Figure 2 and 3) and file 2 (uncropped images of western blot from Figure 2 and 3), or otherwise available from the corresponding author on reasonable request, ChIP-Seq data was deposited

to GEO database with accession number GSE151217 and GSE180367.

Field-specific reporting

Please select the one below that is the best fit for your research. If you are not sure, read the appropriate sections before making your selection.

[x] Life sciences [ ] Behavioural & social sciences [ ] Ecological, evolutionary & environmental sciences

For a reference copy of the document with all sections, see nature.com/documents/nr-reporting-summary-flat.pdf

Life sciences study design

All studies must disclose on these points even when the disclosure is negative.

Sample size	Comparing S309W knock-in mouse skeletal phenotype: wild-type N=8, S309W(WT/Tg) N=1, S309W(Tg/Tg) N=3<br>Osteoblastic differentiation of chondrocytes and MSCs for ALP and Alizarin red staining, experiment replicated 3 times.<br>quantitative real-time PCR for gene expression, N=4-5 for each time point<br>western blot, experiment replicated 5 times with similar results<br>co-IP of SP7 and DLX proteins, experiment replicated 3 times<br>gel mobilitiy shift assay, experiment replicated 3 times
Data exclusions	no data were excluded
Replication	all experimental findings, such as osteoblastic differentiation, qPCR, western blot, co-IP, and gel shift assays, reported in this study were successfully replicated
Randomization	Bone histomorphometric analyses were performed on 4 randomly chosen throughout each bone section
Blinding	for experiments such as qPCR, western blot, co-IP, and gel shift assay, the person performing the experiment did not know the order and identity of the samples.

Reporting for specific materials, systems and methods

We require information from authors about some types of materials, experimental systems and methods used in many studies. Here, indicate whether each material, system or method listed is relevant to your study. If you are not sure if a list item applies to your research, read the appropriate section before selecting a response.

Materials & experimental systems

n/a	Involved in the study	
[ ]	[x]	Antibodies
[ ]	[x]	Eukaryotic cell lines
[x]	[ ]	Palaeontology and archaeology
[ ]	[x]	Animals and other organisms
[ ]	[x]	Human research participants
[ ]	[x]	Clinical data
[x]	[ ]	Dual use research of concern

Methods

n/a	Involved in the study	
[ ]	[x]	ChIP-seq
[x]	[ ]	Flow cytometry
[x]	[ ]	MRI-based neuroimaging

Antibodies

Antibodies used	FLAG antibody (M2, Sigma-Aldrich, 1:1000), myc antibody (TA150121, Origene; ab9106, abcam, 1:1000), HA antibody (ab18181, abcam, 1:1000), Col1a1 (ab34710, abcam, 1:500), Rankl (ab45039, abcam, 1:1000), Gapdh (ab9485, abcam, 1:5000)
Validation	All antibodies are commercially available. For in house validation of epitope tags, we performed western blot comparing cell lysates transfected with tagged proteins or with empty vector. A single discrete band at the expected molecular size indicated that these antibodies can be used to detect FLAG-tagged, HA- tagged, and myc-tagged proteins, respectively.

Eukaryotic cell lines

Policy information about cell lines

Cell line source(s)	Three cell lines were used in the study: HEK293, Phoenix Ecotropic (ECO), and MC3T3-E1.
Authentication	All three cell lines were purchased from ATCC. the cell lines were authenticated by ATCC

Mycoplasma contamination	all cell lines used were tested negative for mycoplasma contamination
Commonly misidentified lines (See ICLAC register)	none

Animals and other organisms

Policy information about studies involving animals; ARRIVE guidelines recommended for reporting animal research

Laboratory animals	For comparing S309W knock-in, C57BL6, both males and females studied at birth. For chondrocytes isolation, C57BL6 males at 7 days old wild-type mice were used. For MSCs isolation, 3-5 weeks old male and female C57BL6 mice were used. All animals obtained from Charles River laboratories.
Wild animals	*Provide details on animals observed in or captured in the field; report species, sex and age where possible. Describe how animals were caught and transported and what happened to captive animals after the study (if killed, explain why and describe method; if released, say where and when) OR state that the study did not involve wild animals.*
Field-collected samples	*For laboratory work with field-collected samples, describe all relevant parameters such as housing, maintenance, temperature, photoperiod and end-of-experiment protocol OR state that the study did not involve samples collected from the field.*
Ethics oversight	All animals were used in accordance with the Guide for the Care and Use of Laboratory Animals (National Research Council 2003).

Note that full information on the approval of the study protocol must also be provided in the manuscript.

Human research participants

Policy information about studies involving human research participants

Population characteristics	This study focuses on a single subject with a complex skeletal disorder including severe scoliosis, thickened calvarium, craniosynostosis, osteosclerosis of the clavicles and spine, and recurring fractures in the lower extremities.
Recruitment	(timeline: born 09/2009, first presentation with plagiocephalus 04/2010, scoliosis 07/2011, long bone fracture09/2011,BONE HISTOLOGY: 03/2013 (age 3.5 yrs),Genetic evaluation starting 05/2012 (age 2.6 yrs, Material sent to NIH lab (around 10/2014).
Ethics oversight	The studies described in this paper were conducted according to the principles of the Declaration of Helsinki, with particular attention to §20/vulnerable groups and individuals. Written consent to perform research on material available from an extensive diagnostic work-up was given by the legal guardians of the patient, in accordance with the Ethical Committee of the Medical University of Vienna. In addition, informed consent was obtained from the legal guardians to publish potentially identifying clinical information including the details of the case, radiographs, and photographs that were included in the final version of the manuscript.

Note that full information on the approval of the study protocol must also be provided in the manuscript.

Clinical data

Policy information about clinical studies

All manuscripts should comply with the ICMJEguidelines for publication of clinical research and a completedCONSORT checklist must be included with all submissions.

Clinical trial registration	The study did not involve a clinical trial
Study protocol	The study did not involve a clinical trial
Data collection	Clinical data and specimens were collected throughout the subjects life, i.e. the 11 years prior to study publications, in Austrian medical centers, especially at the Medical University of Vienna, Vienna, Austria. The Ethical Committee of the medical University of Vienna approved collection of clinical data for research purposes in a rare disease registry of the Vienna Bone and Growth Center.
Outcomes	*Describe how you pre-defined primary and secondary outcome measures and how you assessed these measures.*

ChIP-seq

Data deposition

- [x] Confirm that both raw and final processed data have been deposited in a public database such as GEO.
- [ ] Confirm that you have deposited or provided access to graph files (e.g. BED files) for the called peaks.

Data access links<br>*May remain private before publication.*	https://www.ncbi.nlm.nih.gov/geo/query/acc.cgi?acc=GSE151217<br>https://www.ncbi.nlm.nih.gov/geo/query/acc.cgi?acc=GSE180367

Files in database submission	*Provide a list of all files available in the database submission.*
Genome browser session (e.g. UCSC)	*Provide a link to an anonymized genome browser session for "Initial submission" and "Revised version" documents only, to enable peer review. Write "no longer applicable" for "Final submission" documents.*

Methodology

Replicates	For chondrocytes, no technical replicate, biological samples were each pooled from 3 retroviral infections.<br>For MC3T3, there were both technical replicate and biological replicate. Cells from 2 transfection were pooled and the whole experiment was repeated.
Sequencing depth	For input DNA, total number of reads: 42673288; uniquely mapped reads: 13225574. For FLAG wild-type SP7 ChIP, total number of reads: 50696492; uniquely mapped reads: 16055743. For FLAG mutant SP7 ChIP, total number of reads: 88259526; uniquely mapped reads: 39045400. All samples are 150 bp sequences, paired-end.
Antibodies	FLAG M2 antibody (Sigma, F1804)
Peak calling parameters	Reads were aligned to the mm9 genome assembly using bowtie 1.2.2 with follwing options: -v 2 -m 1. Peak calling was performed by two-sample analysis on CisGenome software with a P-value cutoff of 10-5 comparing with the input control
Data quality	All the peaks obtained are FDR <0.01
Software	GREAT GO analysis was performed utilizing the on line GREAT GO program, version 4.0.4. Each peak category was run against a whole-genome background with assembly mm9. De novo motif analyses were performed using DREME; a 100 bp region surrounding peak center was extracted from mm9 and used for the analysis. Peak intersection was performed by BEDTools-Version-2.16.2.
