# Supplementary material for: A neomorphic variant in SP7 alters sequence specificity and causes a high-turnover bone disorder
Source: Nat Commun. 2022 Feb 4;13:700. doi: 10.1038/s41467-022-28318-4 (PMC8816926; doi:10.1038/s41467-022-28318-4)
Supplement: Supplementary file 4 — Description of Additional Supplementary Files [file 41467_2022_28318_MOESM4_ESM.pdf]

**Title:** Supplemental Movie 1.
**Description:** Movie showing three-dimensional whole skeleton micro-CT imaging of newborn wild-type mouse.

**Title:** Supplemental Movie 2.
**Description:** showing three-dimensional whole skeleton micro-CT imaging of newborn S309W heterozygous knock-in mouse.

**Title:** Supplemental Movie 3.
**Description:** Movie showing three-dimensional whole skeleton micro-CT imaging of newborn S309W homozygous knock-in mouse.

**Title:** Supplemental Movie 4.
**Description:** Movie showing three-dimensional whole skeleton micro-CT imaging of 3 weeks old wild-type mouse.

**Title:** Supplemental Movie 5.
**Description:** Movie showing three-dimensional whole skeleton micro-CT imaging of 3 weeks old S309W mosaic mouse.

**Title:** Supplemental Movie 6.
**Description:** Movie showing three-dimensional whole skeleton micro-CT imaging of 20 weeks old wild-type mouse.

**Title:** Supplemental Movie 7.
**Description:** Movie showing three-dimensional whole skeleton micro-CT imaging of 20 weeks old S309W mosaic mouse.

**Title:** Supplemental Data 1.
**Description:** Summary of mice generated using CRISPR Cas9.

**Title:** Supplemental Data 2.
**Description:** Summary of genotype and phenotype of the four S309W mosaic mice.

**Title:** Supplemental Data 3.
**Description:** Comparison between SP7-associated high turnover bone disorder and other forms of high turnover disorders.
