# Supplementary material for: A neomorphic variant in SP7 alters sequence specificity and causes a high-turnover bone disorder
Source: Nat Commun. 2022 Feb 4;13:700. doi: 10.1038/s41467-022-28318-4 (PMC8816926; doi:10.1038/s41467-022-28318-4)
Supplement: Supplementary file 15 — Source Data File [file 41467_2022_28318_MOESM15_ESM.zip › source data 2 (markers labeled).pptx]

## Slide 1
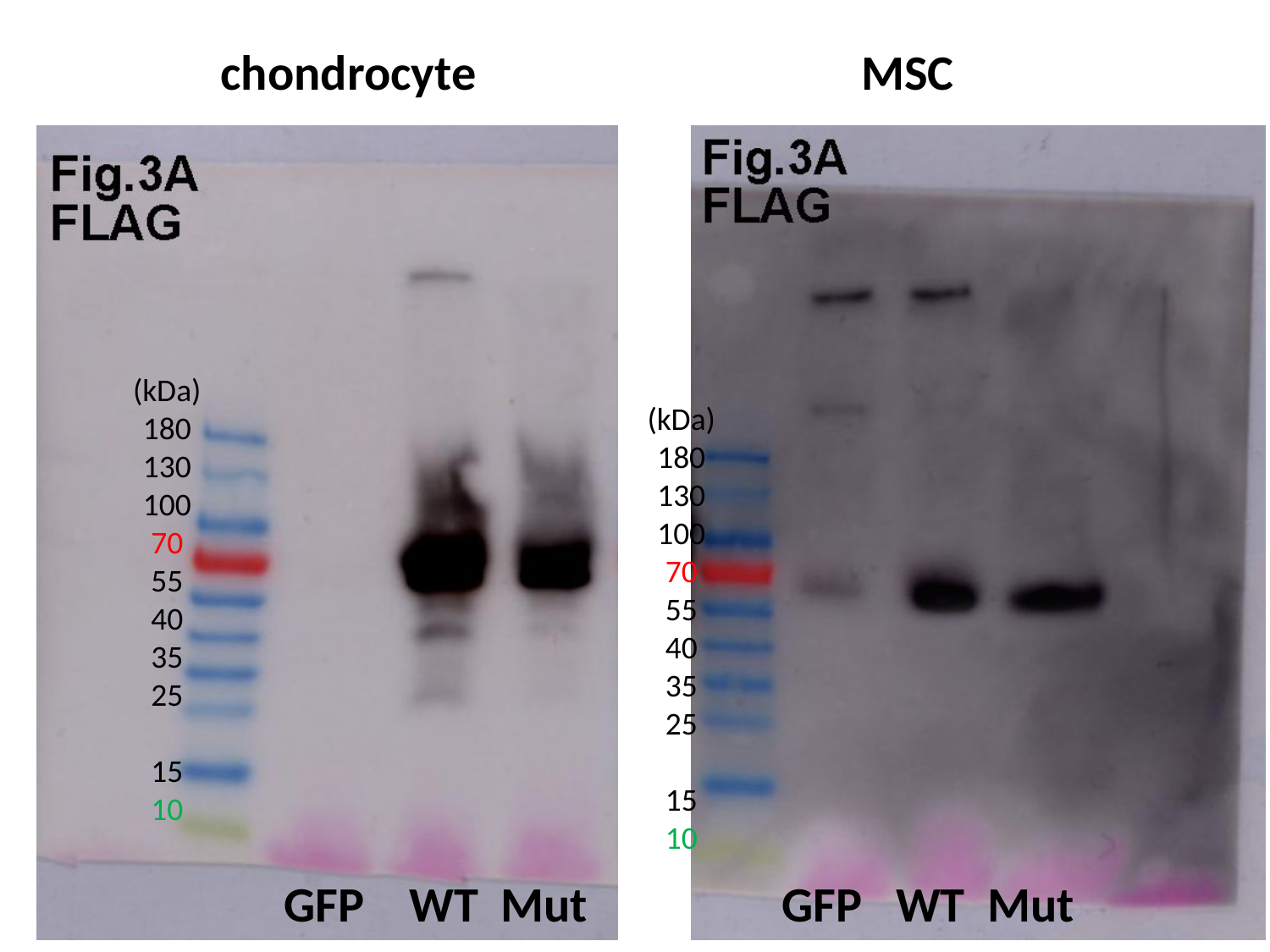

chondrocyte
MSC
(kDa)
180
130
100
70
55
40
35
25
15
10
(kDa)
180
130
100
70
55
40
35
25
15
10
GFP WT Mut
GFP WT Mut

## Slide 2
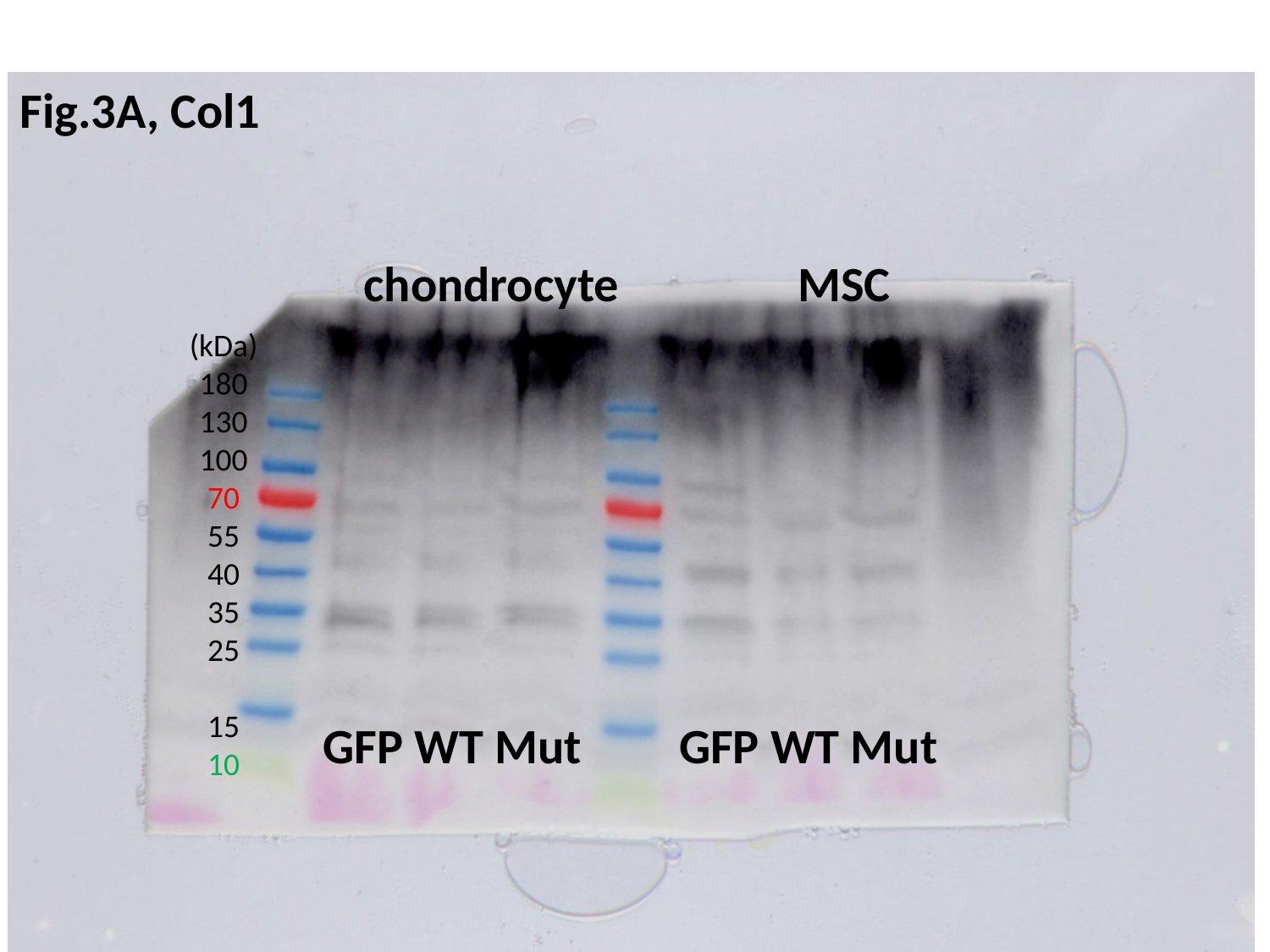

Fig.3A, Col1
chondrocyte
MSC
(kDa)
180
130
100
70
55
40
35
25
15
10
GFP WT Mut
GFP WT Mut

## Slide 3
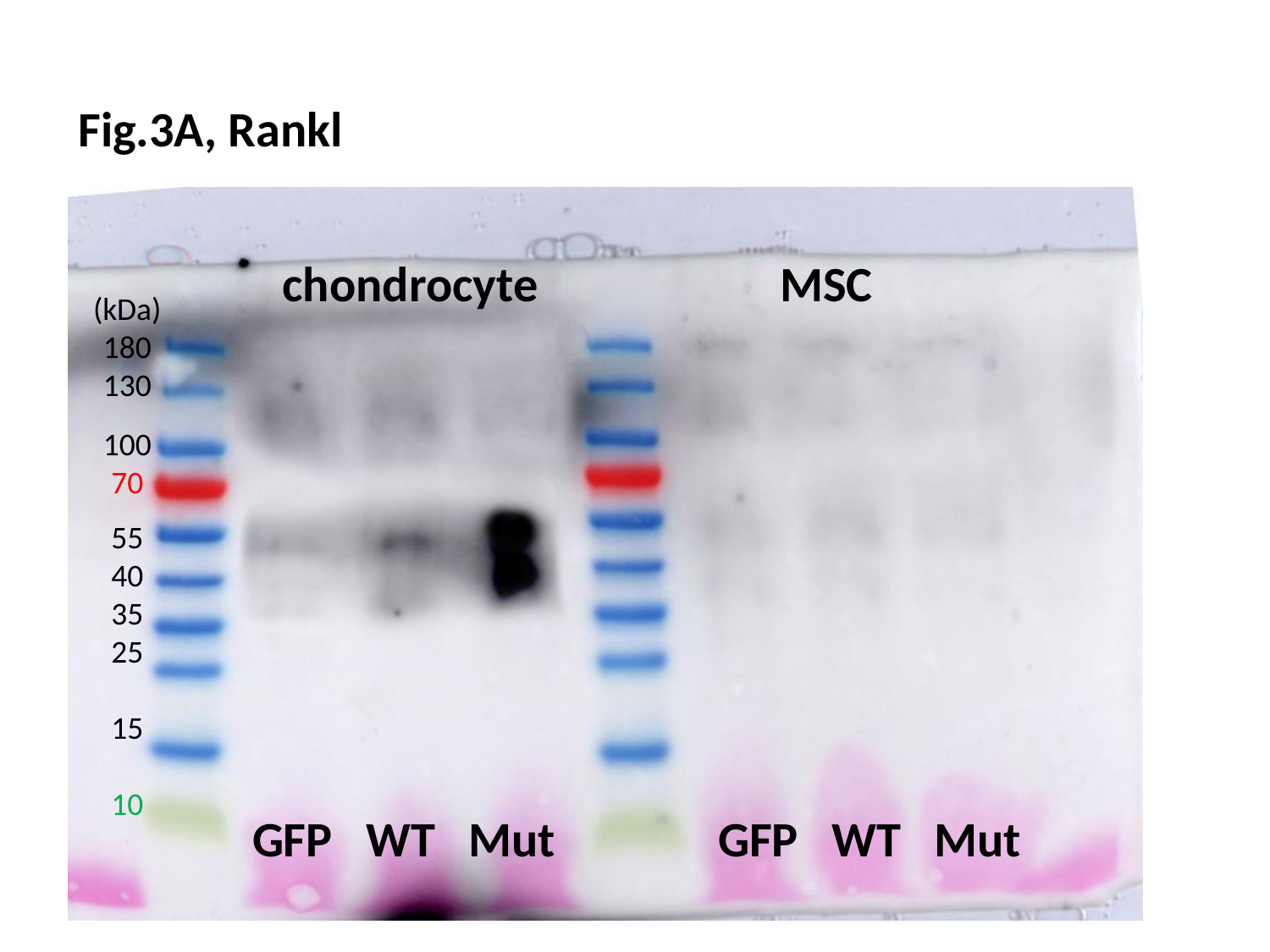

Fig.3A, Rankl
chondrocyte
MSC
(kDa)
180
130
100
70
55
40
35
25
15
10
GFP WT Mut
GFP WT Mut

## Slide 4
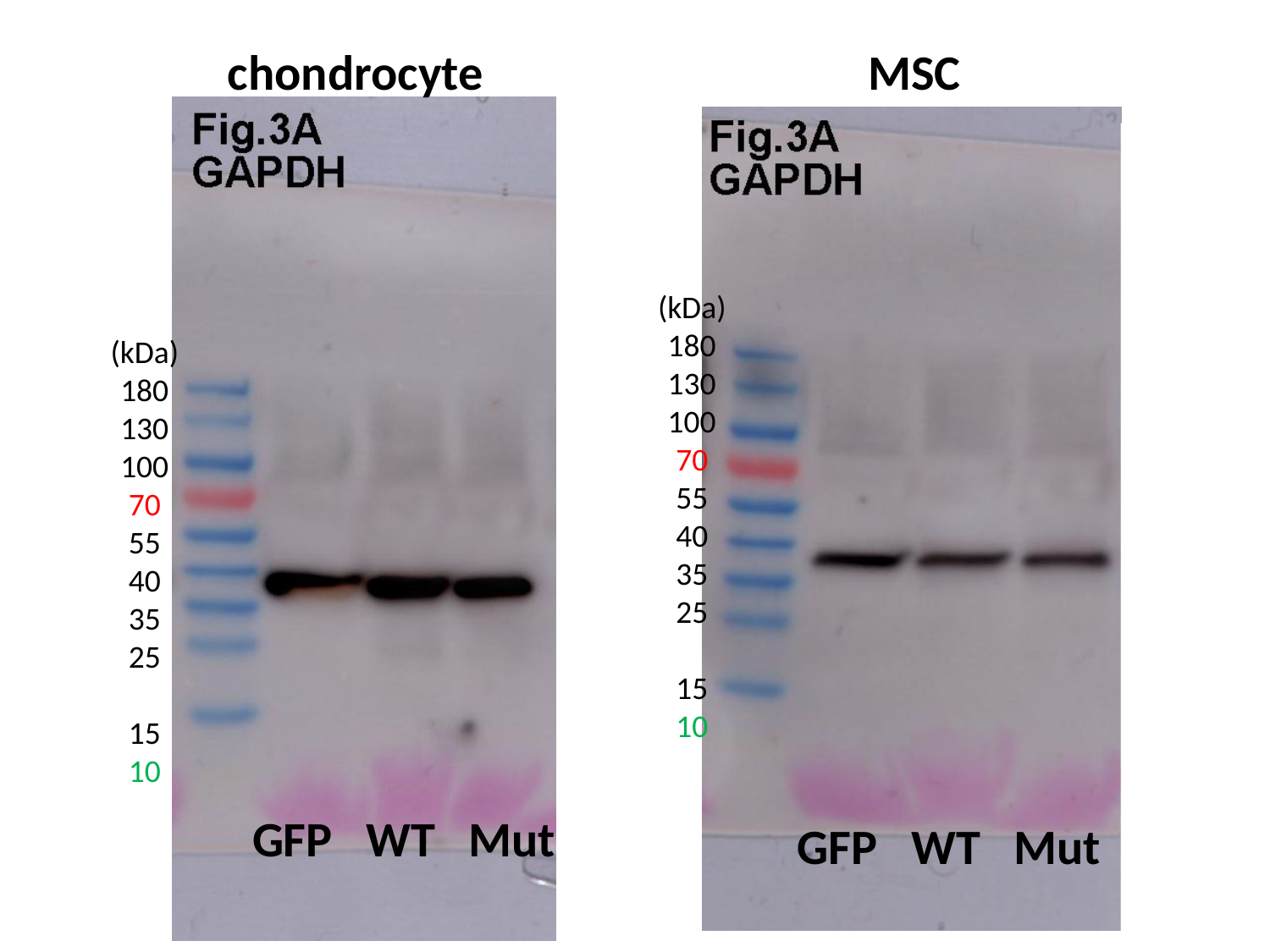

chondrocyte
MSC
(kDa)
180
130
100
70
55
40
35
25
15
10
(kDa)
180
130
100
70
55
40
35
25
15
10
GFP WT Mut
GFP WT Mut

## Slide 5
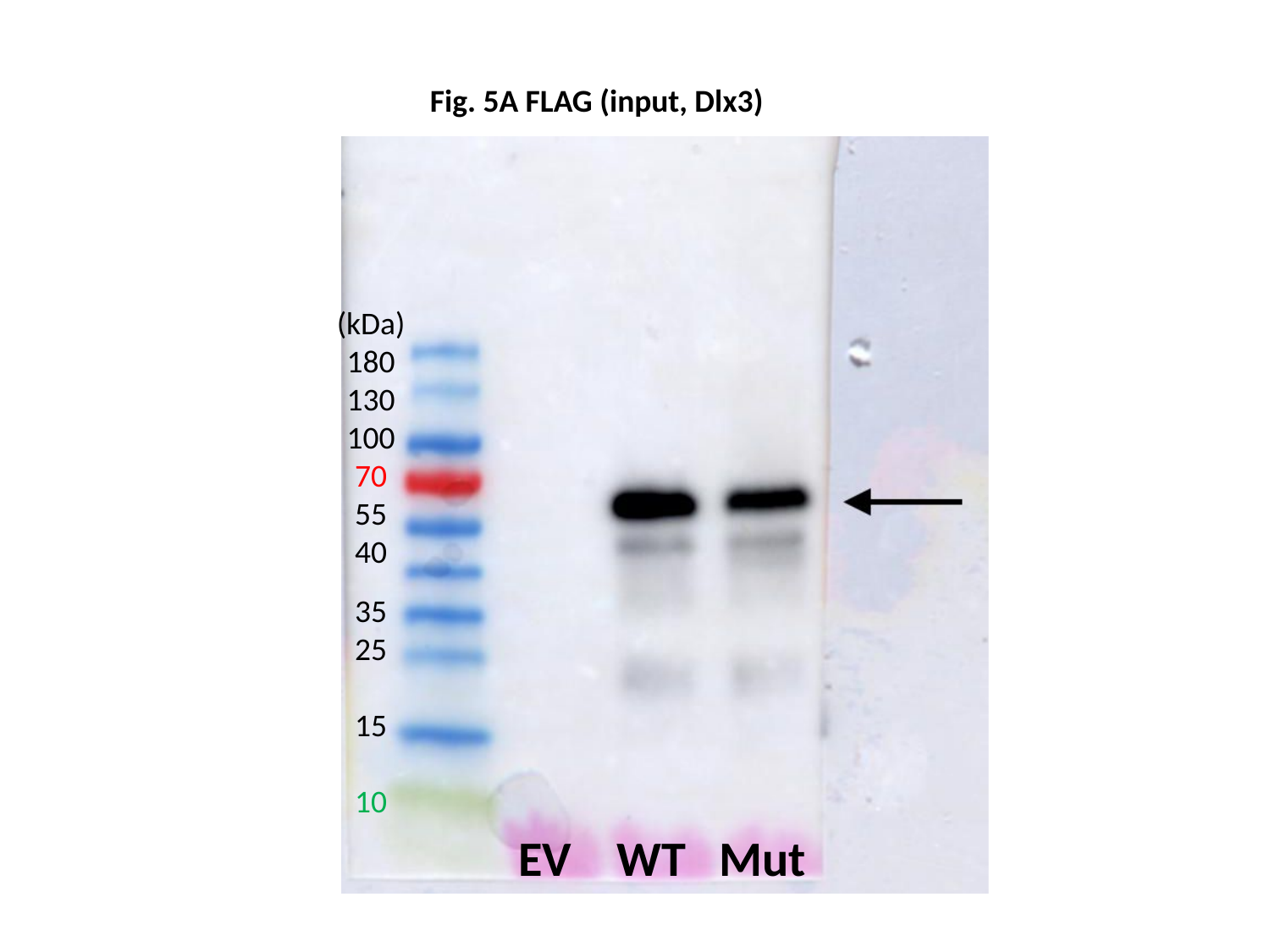

Fig. 5A FLAG (input, Dlx3)
(kDa)
180
130
100
70
55
40
35
25
15
10
 EV WT Mut

## Slide 6
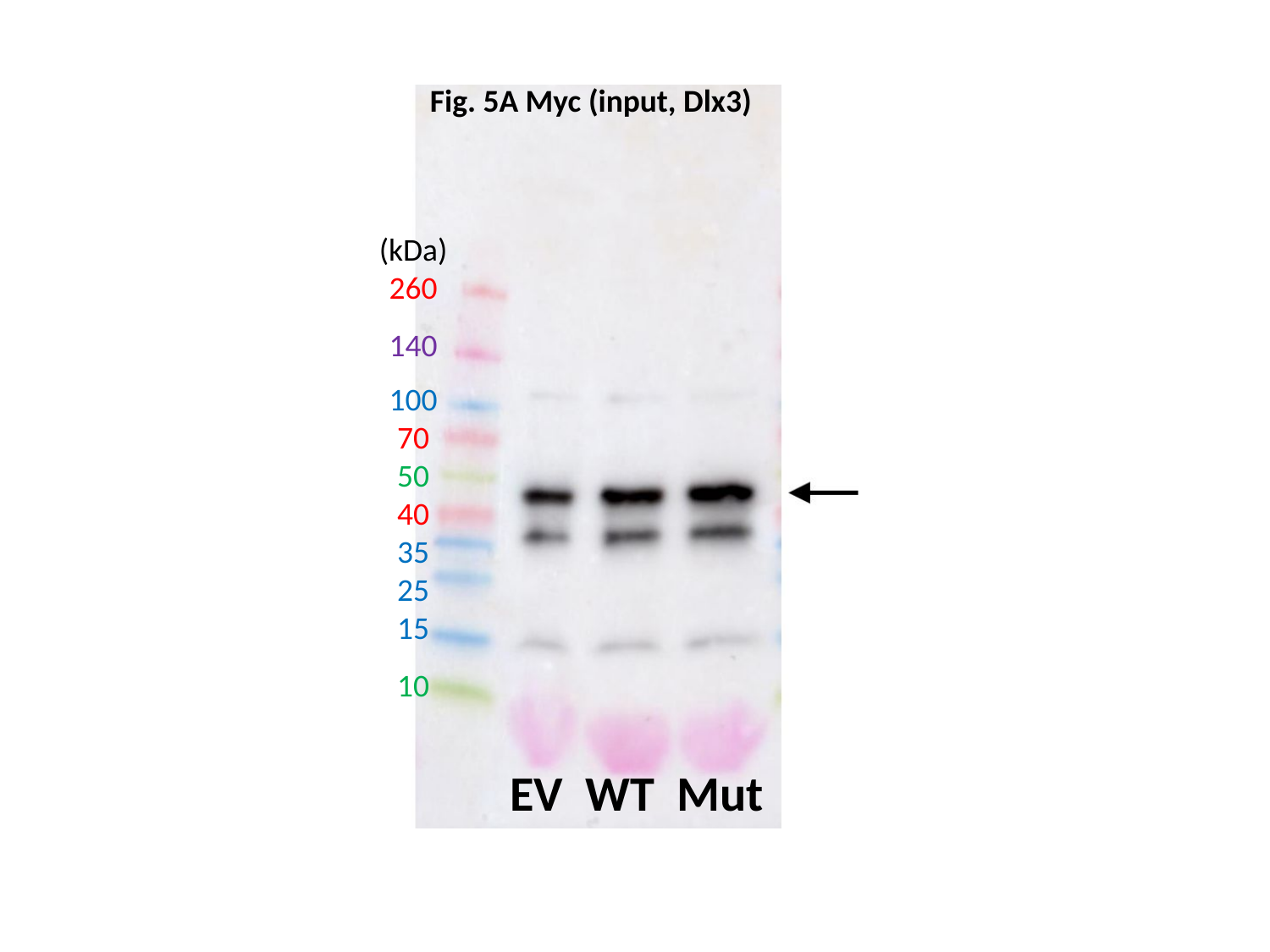

Fig. 5A Myc (input, Dlx3)
(kDa)
260
140
100
70
50
40
35
25
15
10
EV WT Mut

## Slide 7
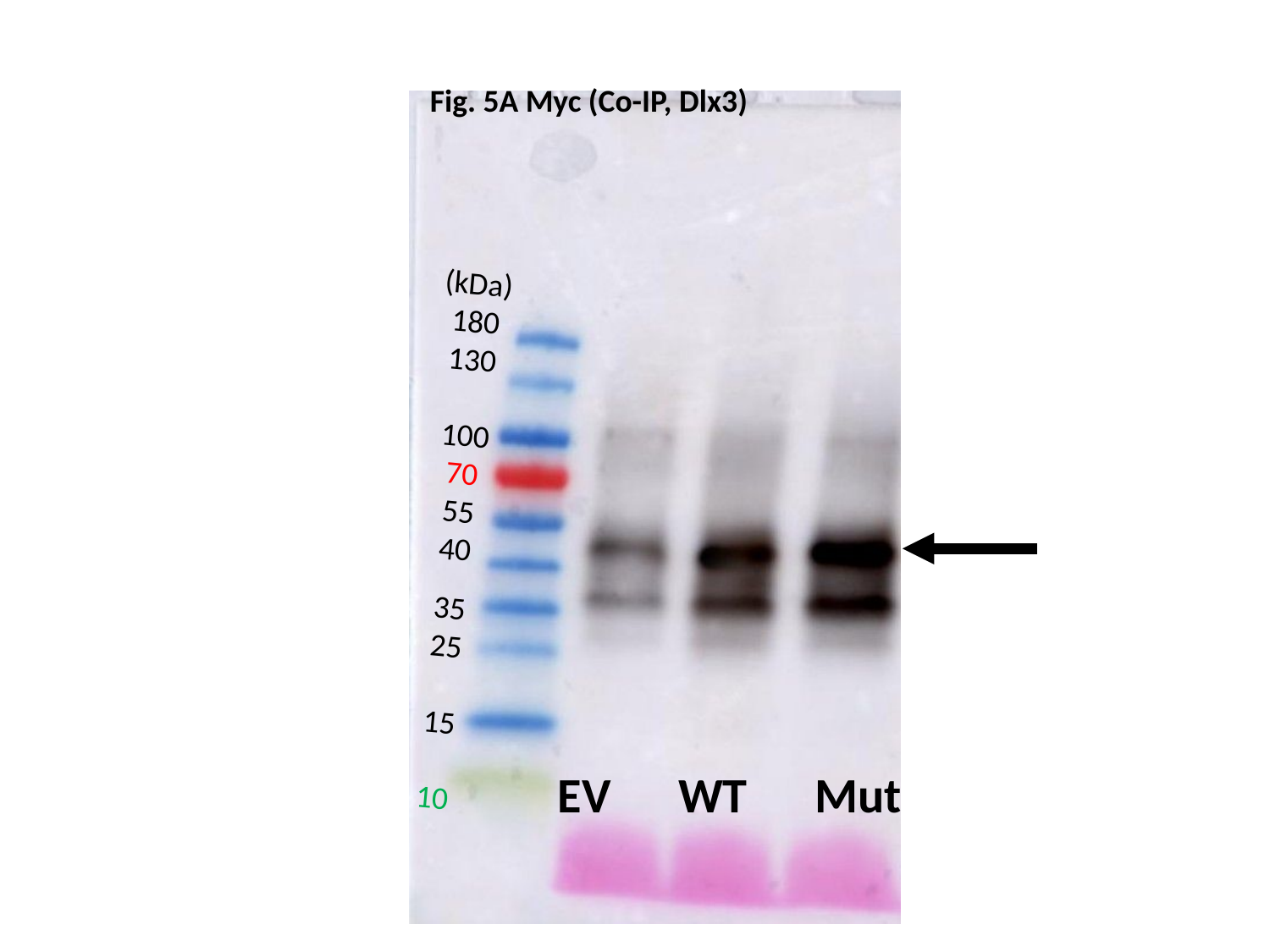

Fig. 5A Myc (Co-IP, Dlx3)
(kDa)
180
130
100
70
55
40
35
25
15
10
EV WT Mut

## Slide 8
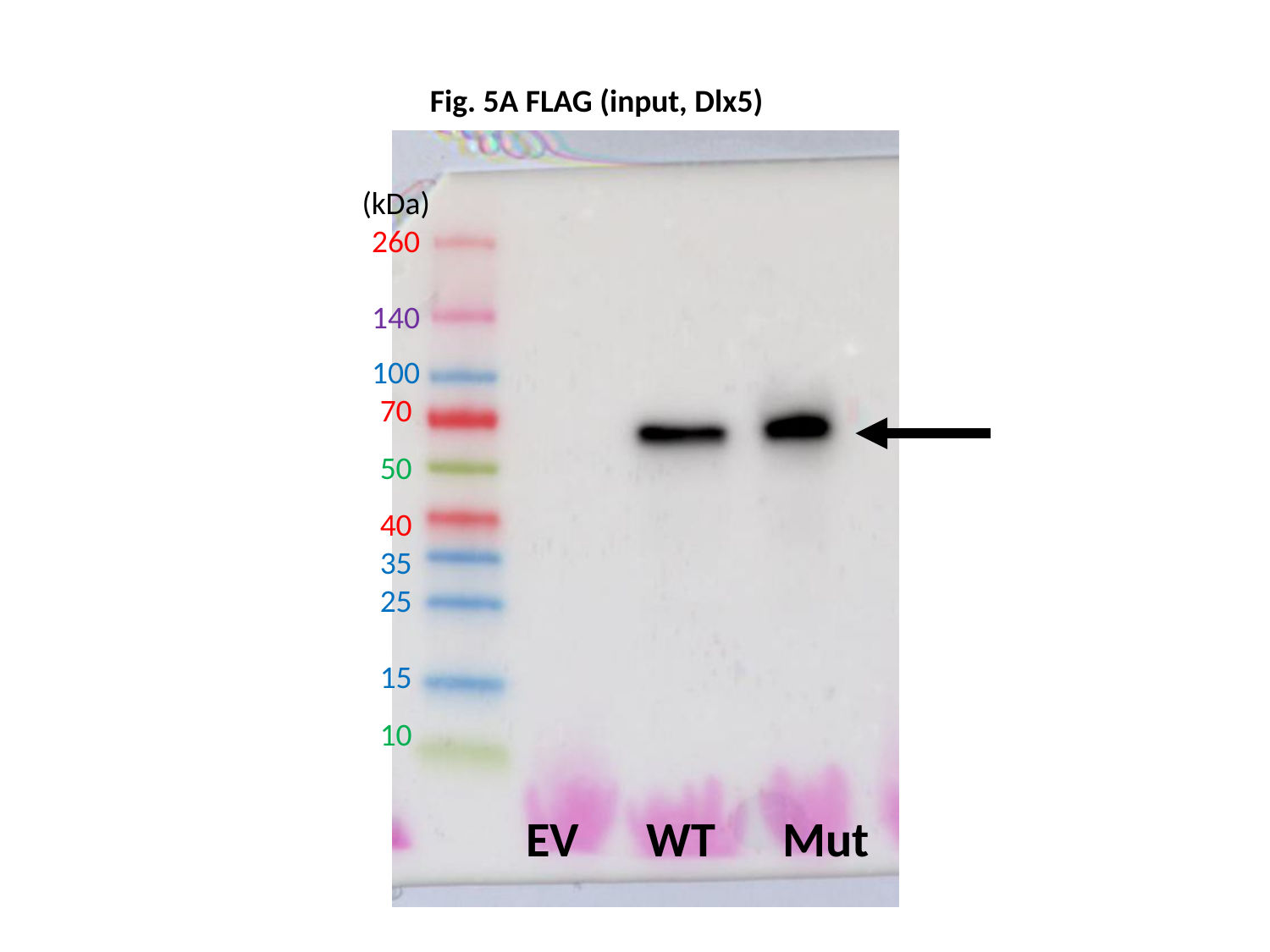

Fig. 5A FLAG (input, Dlx5)
(kDa)
260
140
100
70
50
40
35
25
15
10
EV WT Mut

## Slide 9
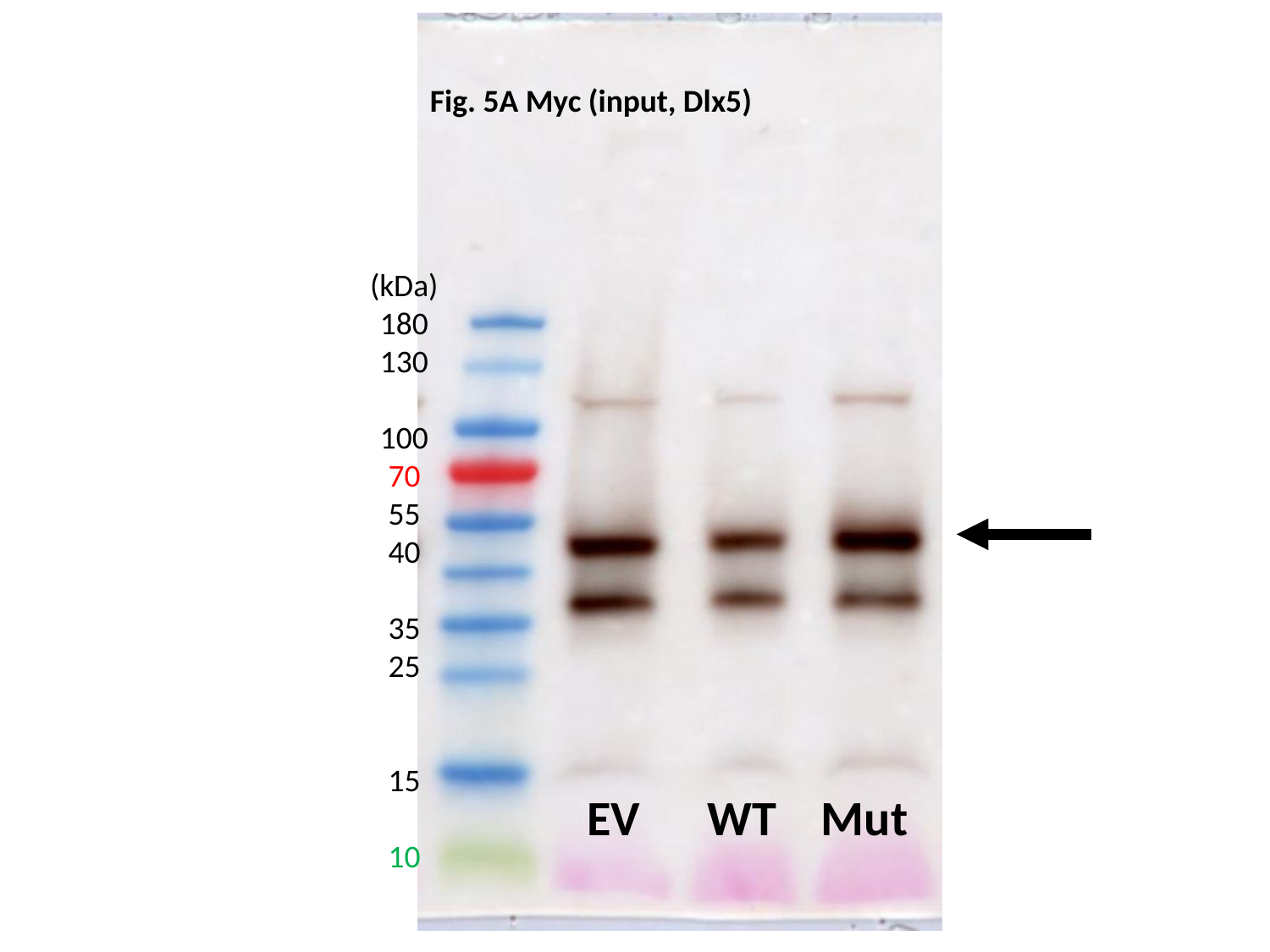

Fig. 5A Myc (input, Dlx5)
(kDa)
180
130
100
70
55
40
35
25
15
10
EV WT Mut

## Slide 10
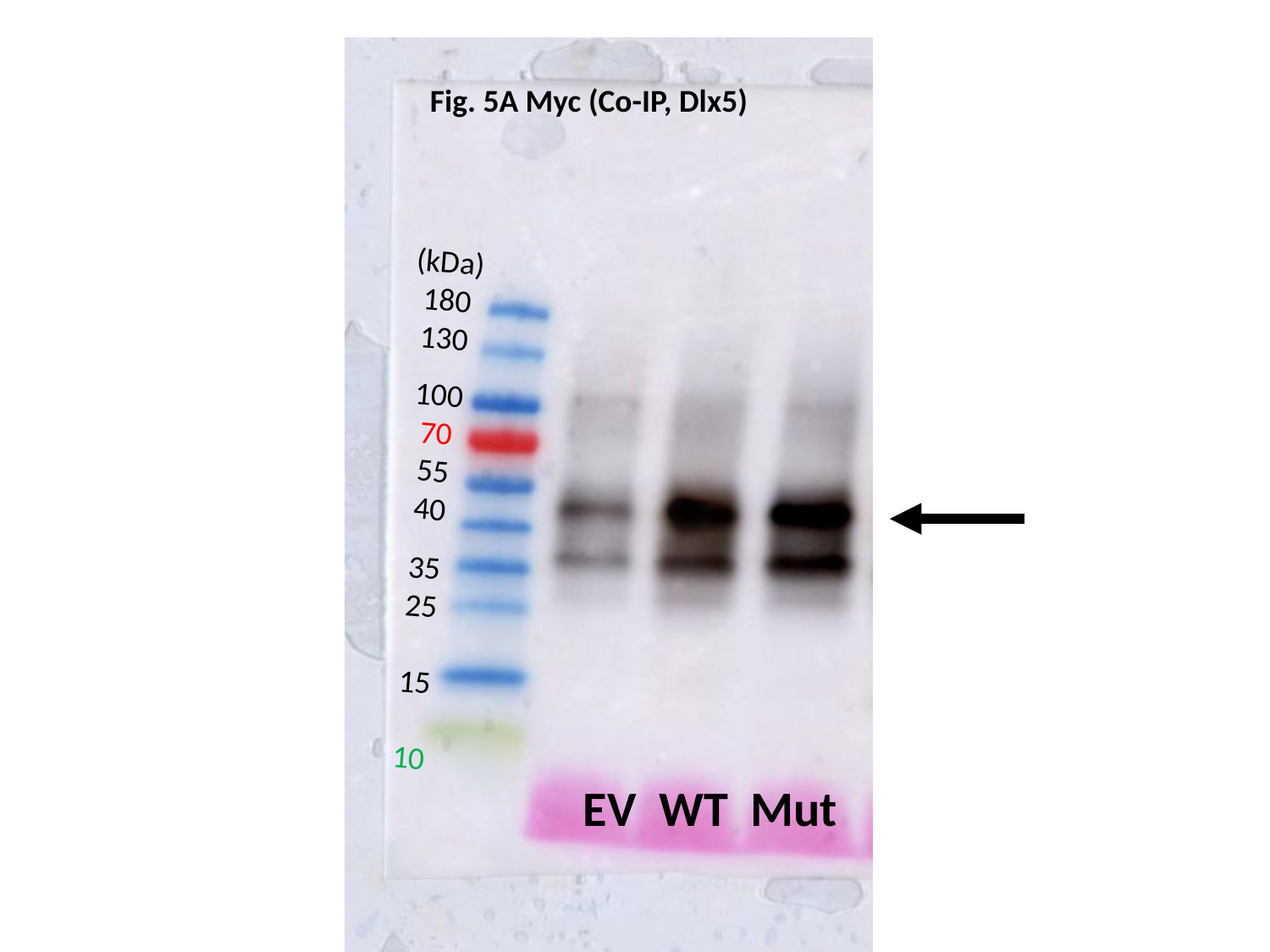

Fig. 5A Myc (Co-IP, Dlx5)
(kDa)
180
130
100
70
55
40
35
25
15
10
EV WT Mut

## Slide 11
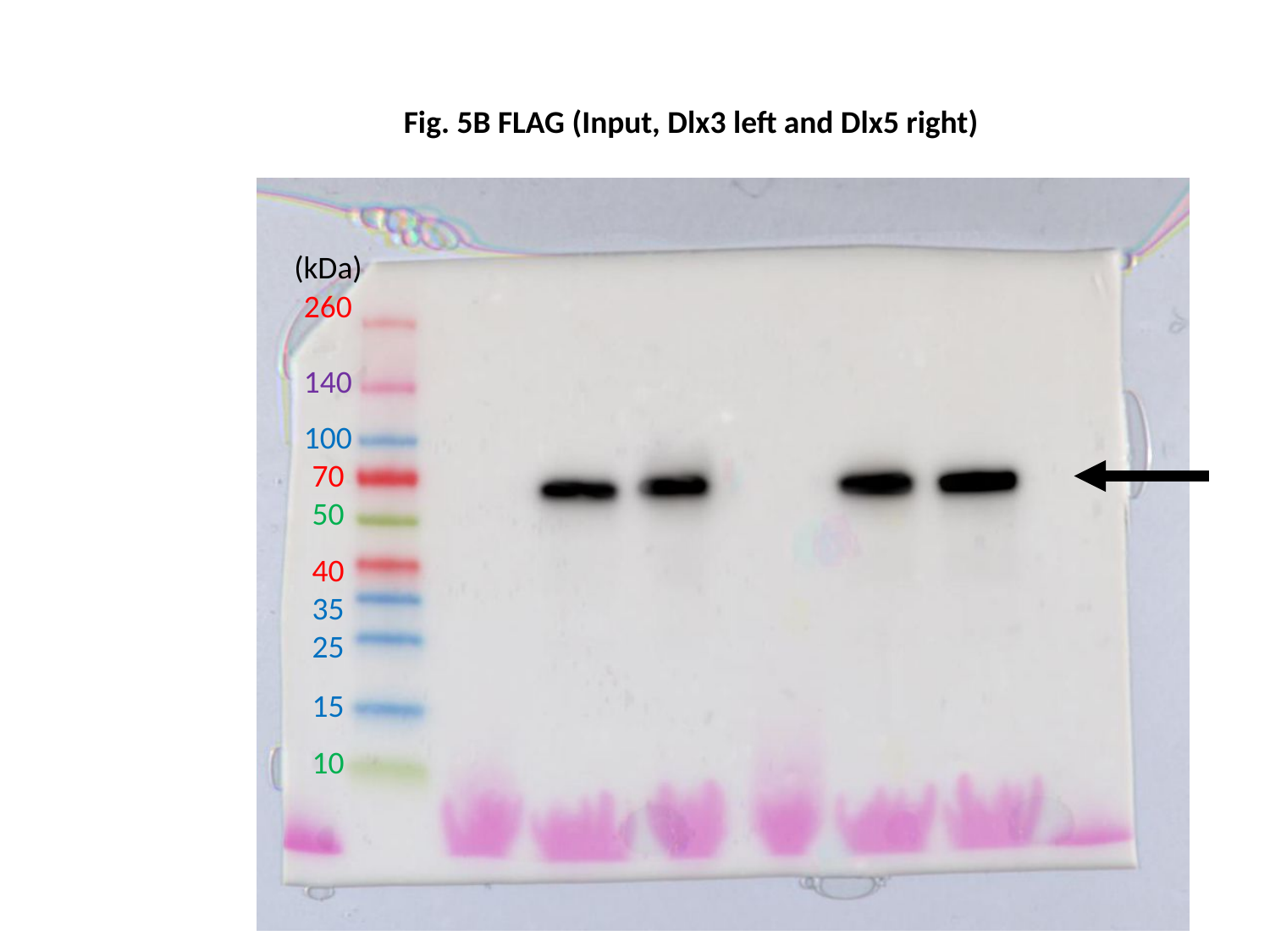

Fig. 5B FLAG (Input, Dlx3 left and Dlx5 right)
(kDa)
260
140
100
70
50
40
35
25
15
10

## Slide 12
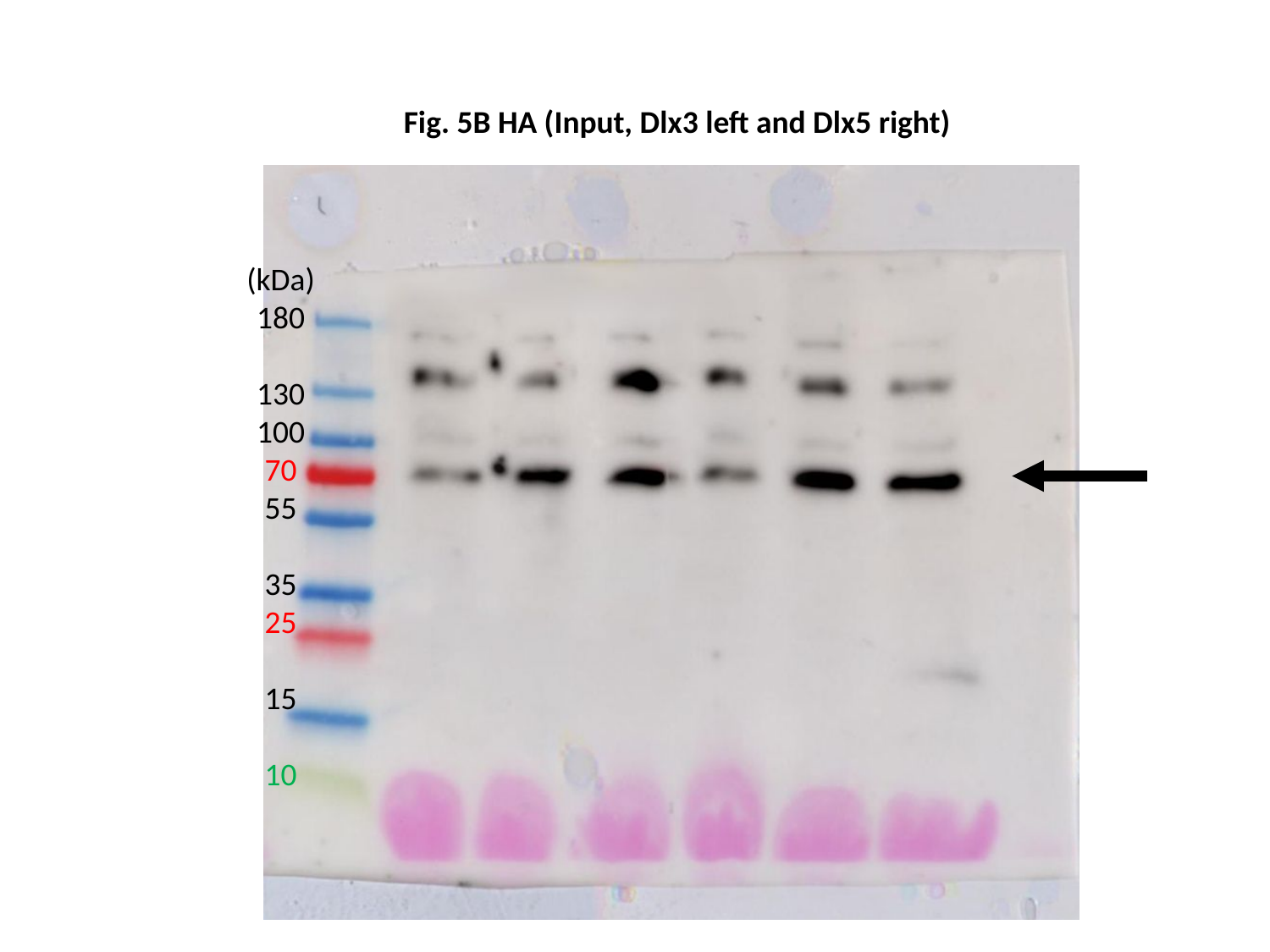

Fig. 5B HA (Input, Dlx3 left and Dlx5 right)
(kDa)
180
130
100
70
55
35
25
15
10

## Slide 13
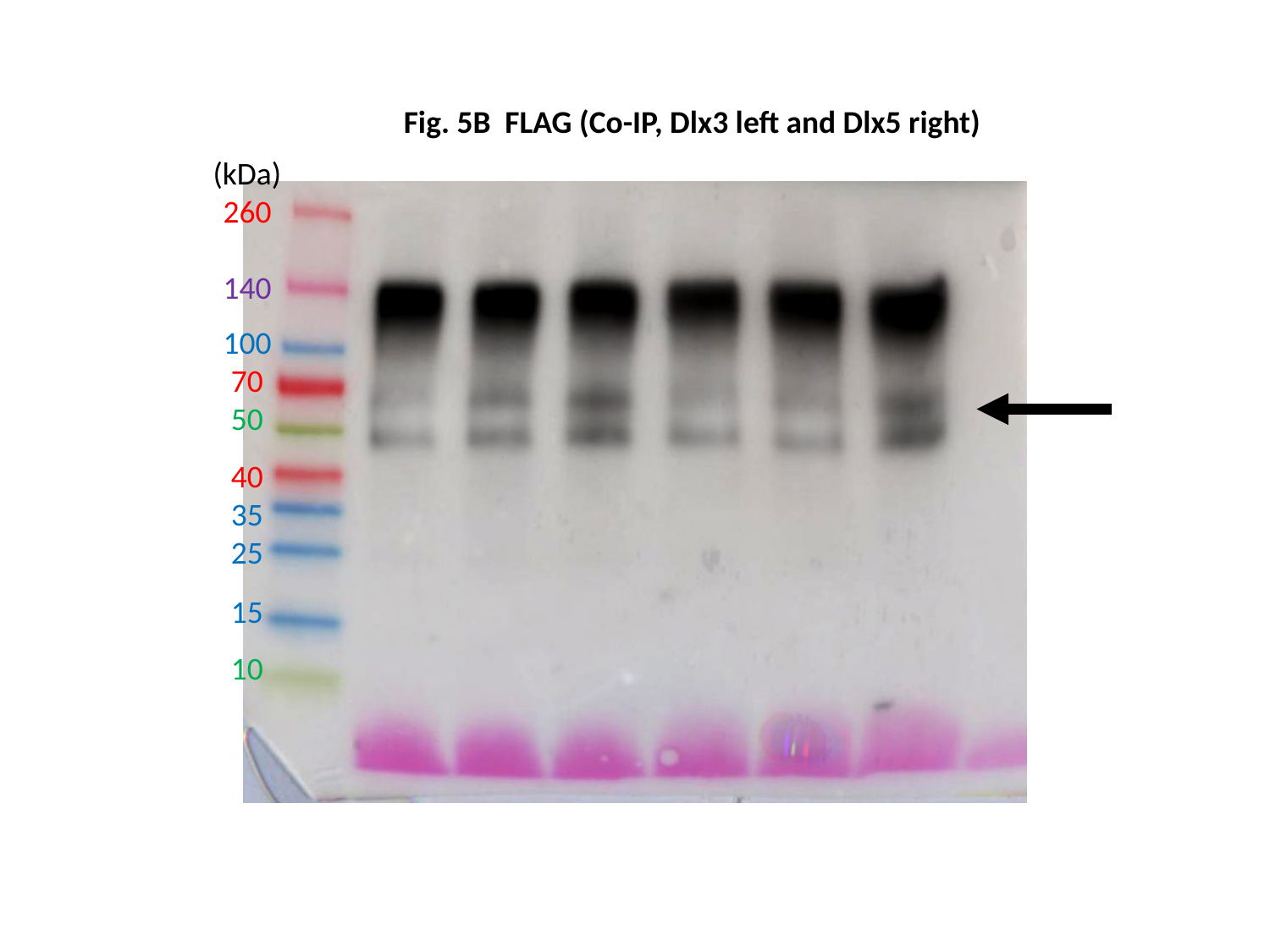

Fig. 5B FLAG (Co-IP, Dlx3 left and Dlx5 right)
(kDa)
260
140
100
70
50
40
35
25
15
10

## Slide 14
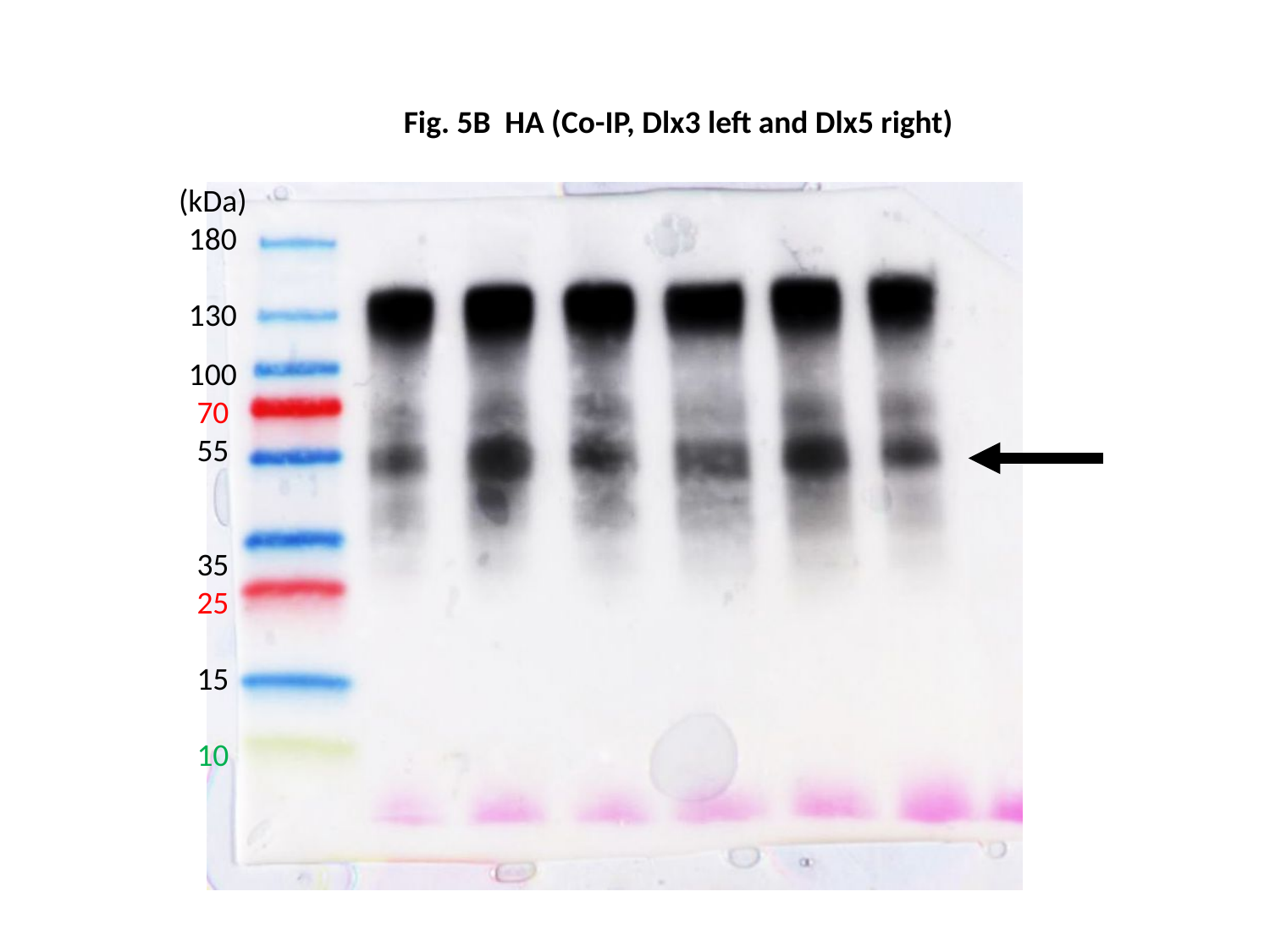

Fig. 5B HA (Co-IP, Dlx3 left and Dlx5 right)
(kDa)
180
130
100
70
55
35
25
15
10

## Slide 15
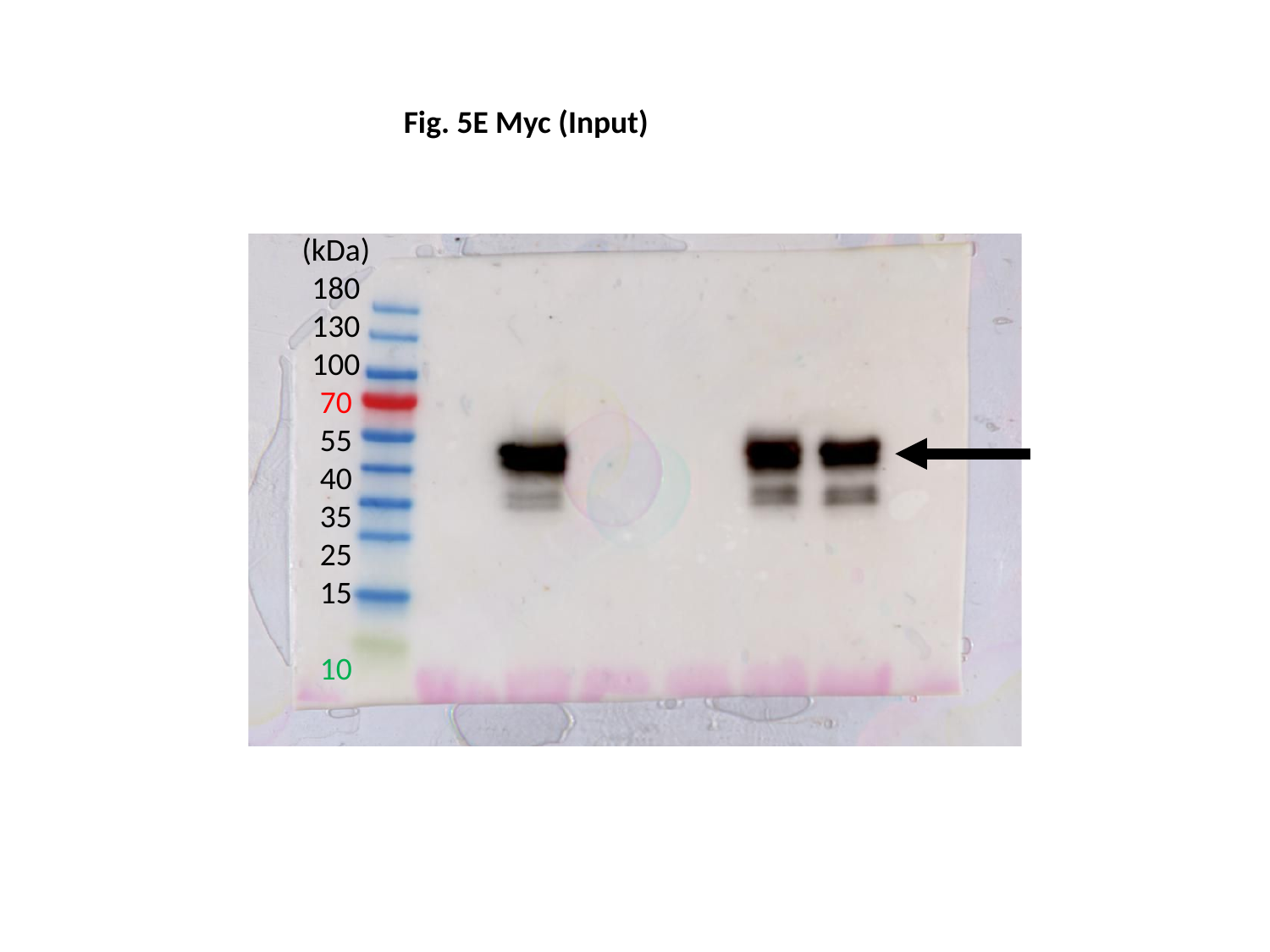

Fig. 5E Myc (Input)
(kDa)
180
130
100
70
55
40
35
25
15
10

## Slide 16
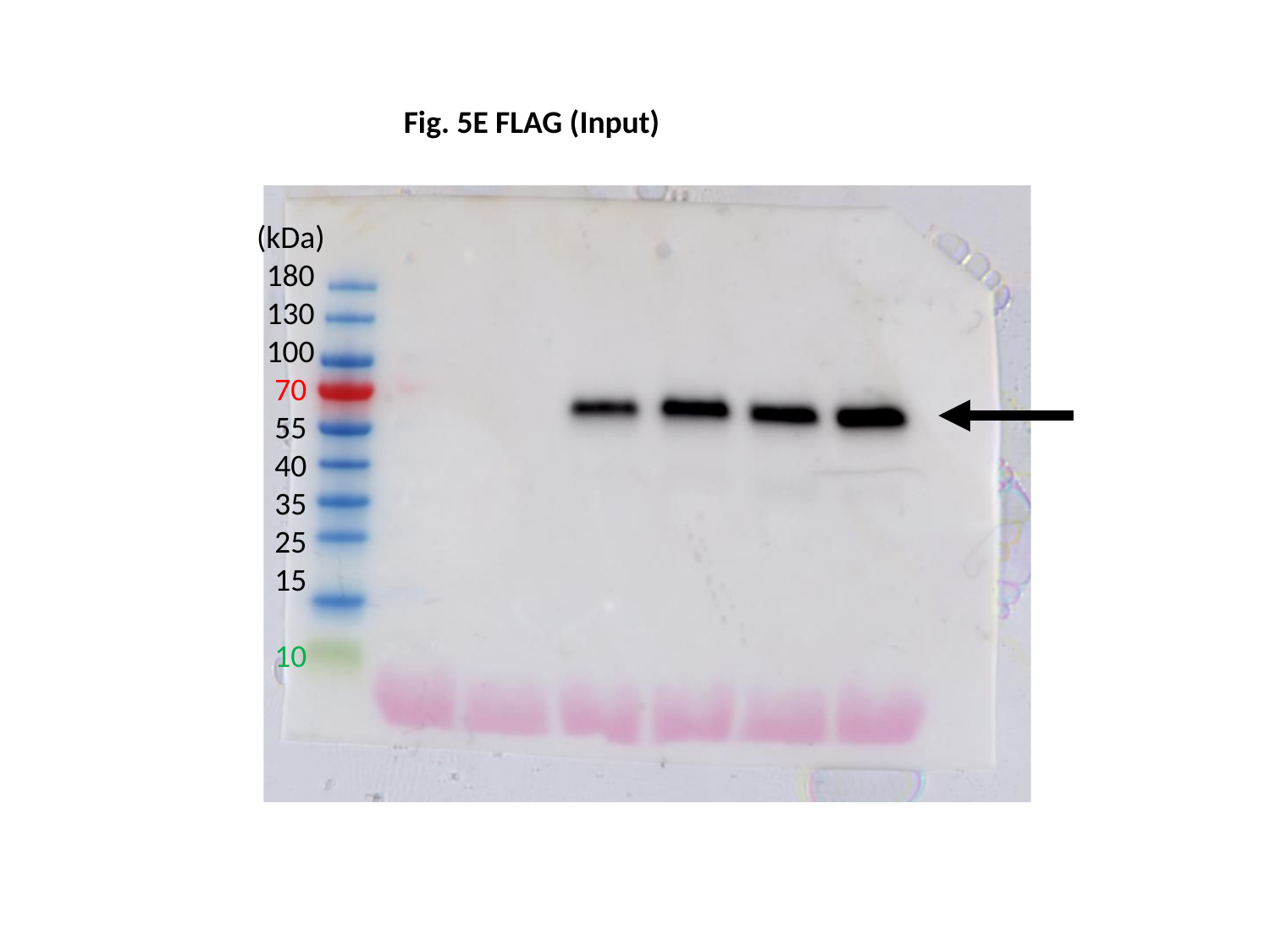

Fig. 5E FLAG (Input)
(kDa)
180
130
100
70
55
40
35
25
15
10

## Slide 17
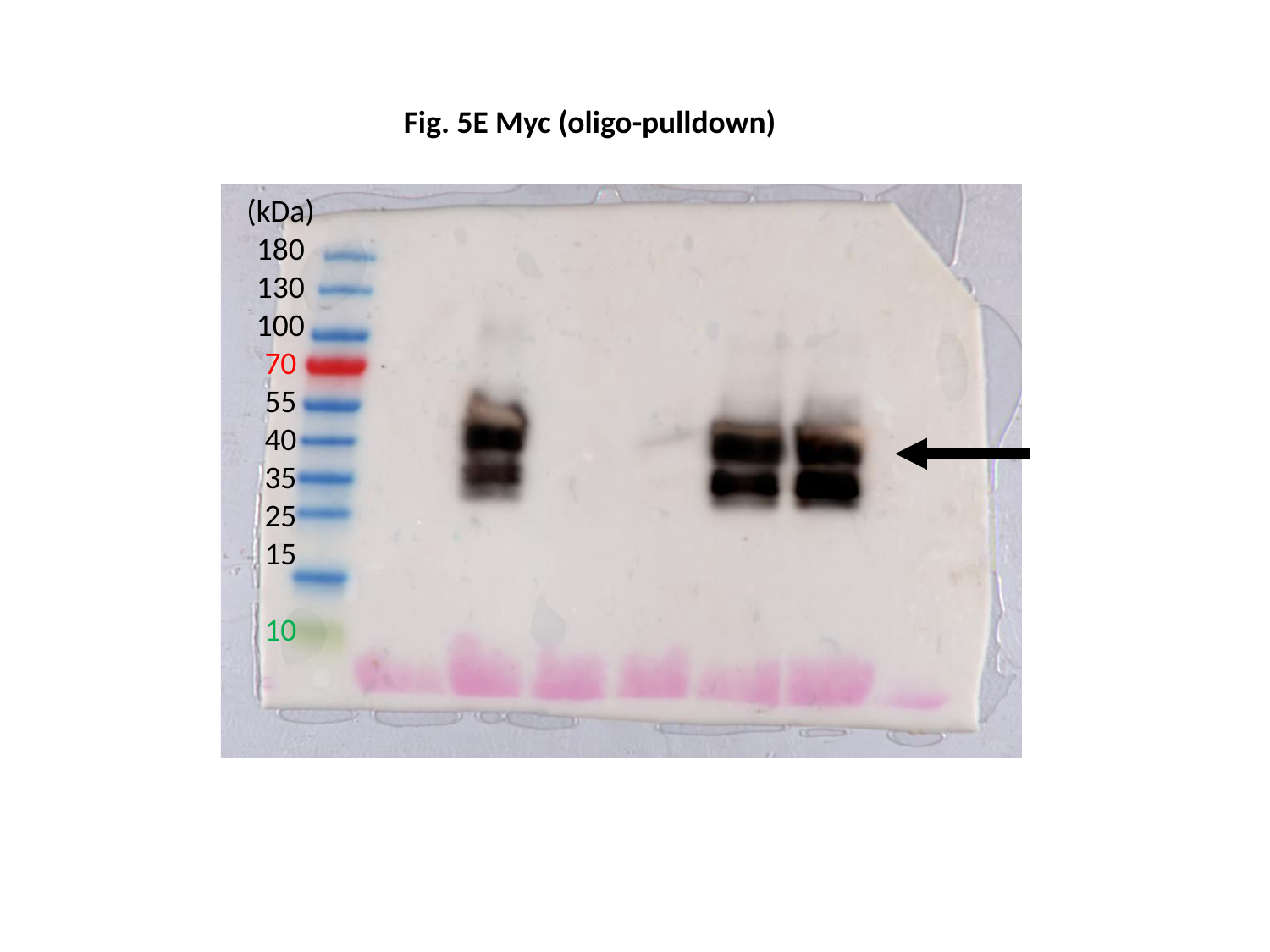

Fig. 5E Myc (oligo-pulldown)
(kDa)
180
130
100
70
55
40
35
25
15
10

## Slide 18
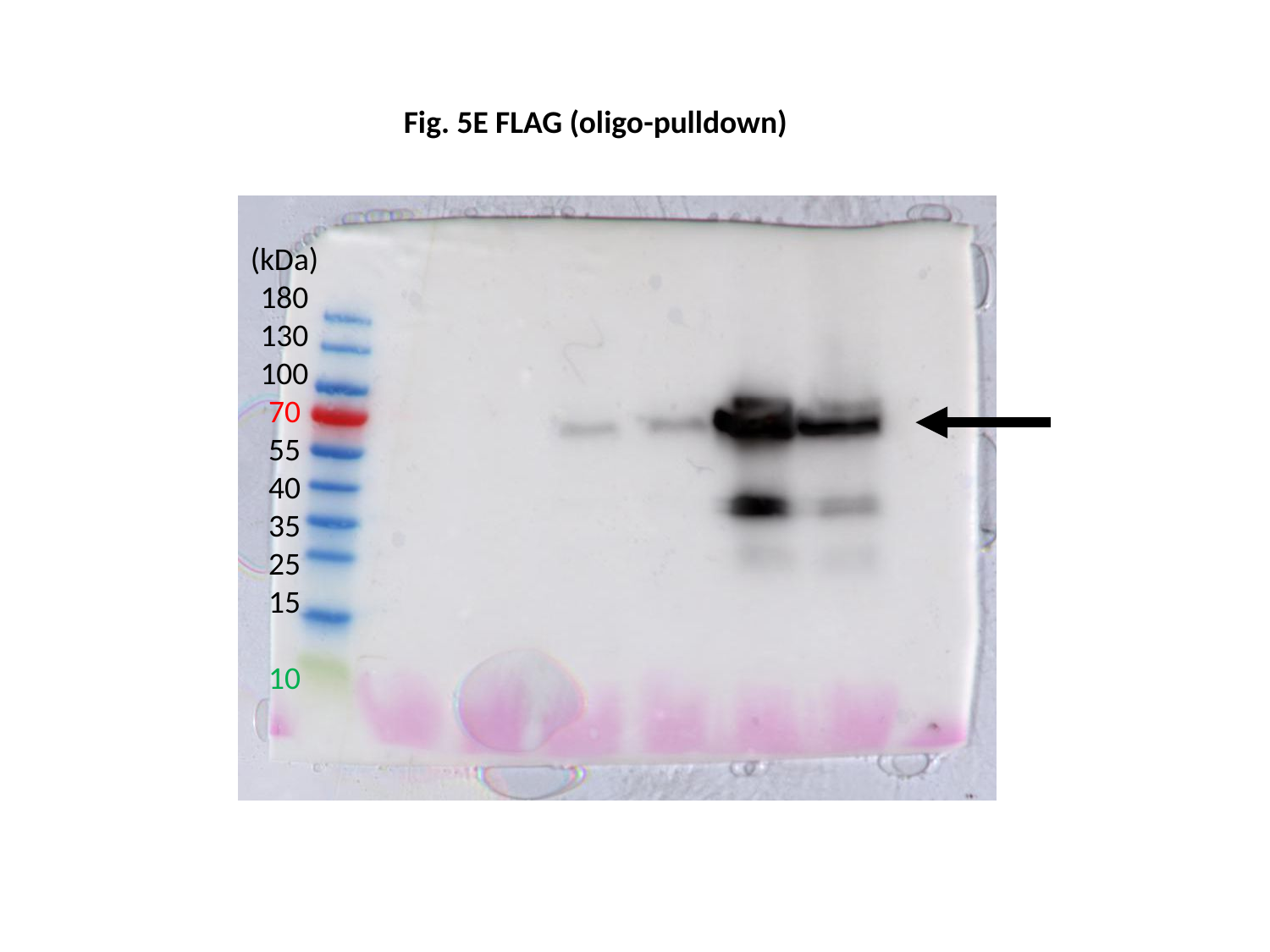

Fig. 5E FLAG (oligo-pulldown)
(kDa)
180
130
100
70
55
40
35
25
15
10
